# Neighborhood collective efficacy and children and adolescents’ externalizing behaviors across development: A systematic review

**DOI:** 10.1371/journal.pone.0337512

**Published:** 2026-01-23

**Authors:** Jiye Lee, Yui Matsuda, Daniel S. Messinger, Thomas M. O’Shea, Yue Pan, Hudson P. Santos Jr

**Affiliations:** 1 School of Nursing and Health Studies, University of Miami, Coral Gables, Florida, United States of America; 2 Department of Psychology, Pediatrics, Electrical & Computer Engineering, and Music Engineering, University of Miami, Coral Gables, Florida, United States of America; 3 Department of Pediatrics, University of North Carolina-Chapel Hill, North Carolina, United States of America; 4 Department of Public Health Sciences, Miller School of Medicine, University of Miami, Miami, Florida, United States of America; Texas Christian University, UNITED STATES OF AMERICA

## Abstract

**Objective:**

To synthesize and describe the relationship between neighborhood collective efficacy (NCE) and children and adolescents’ externalizing behaviors to inform practice and policy decisions.

**Study design:**

Data sources including PubMed, PsycINFO, and CINAHL were searched in November 2024 using the PRISMA guidelines. Literature that addressed the main predictor (neighborhood collective efficacy) and outcome (child externalizing behaviors) were included. Two authors independently evaluated the studies using the guidelines from the National Heart, Lung, and Blood Institute (NHBLI) Quality Assessment Tool for Observational Cohort and Cross-sectional studies. We developed an extraction table to categorize and analyze each study.

**Results:**

We screened 294 abstracts and included 17 studies with a total of 28,957 caregiver-child (or children) dyads and 592 adolescents in November 2024 via database searches through PubMed, PsycINFO, and CINAHL. Consistent with previous literature highlighting the importance of neighborhood environment on child behavioral health outcomes, most studies demonstrated significant relationship between neighborhood collective efficacy and child externalizing behaviors across diverse developmental periods. Furthermore, studies focusing on early childhood yielded the most consistent evidence for the relationship between neighborhood collective efficacy and externalizing behaviors as compared to studies of older developmental periods. In addition, studies resulting from US-based participants were more likely to be significant than studies in other contexts. We also found limited evidence for mediating effects of corporal punishment, parenting, and adverse childhood experiences between neighborhood collective efficacy and child externalizing behaviors.

**Conclusions:**

There is a significant inverse relationship between neighborhood collective efficacy and child externalizing behaviors across diverse developmental stages, populations, and study approaches.

## Introduction

Childhood mental and behavioral disorders have been on the rise in the United States (US) in recent years. Recent statistics released by the Centers for Disease Control and Prevention on youth mental health demonstrates that 20.9% of adolescents aged 12–17 years in the US have experienced a major depressive episode, and 36.7% of high school students reported persistently feeling sad or hopeless in the past year, putting them at a heightened risk for developing a mental health condition [1,2]. Furthermore, suicide continues to be one of the leading causes of mortality in adolescence [1]. Reflecting the magnitude of the mental health crisis, pediatric mental health hospitalizations increased by 25.8% nationwide from 2009 to 2019 [3]. Due to these concerning national trends, the US Surgeon General issued a Surgeon General’s Advisory on protecting youth mental health, and the American Academy of Child and Adolescent Psychiatry, the American Academy of Pediatrics, and the Children’s Hospital Association jointly declared a National State of Emergency in children’s mental health crisis in 2021 [4,5].

### Childhood externalizing behaviors

Childhood externalizing behaviors include negative behaviors, such as aggression, defiance, inattention, hyperactivity, disruption, hostility, and poor impulse control, stemming from a lack of behavior regulation [6,7]. These behaviors are some of the most common reasons for referral to child and adolescent mental health services [8,9]. Addressing externalizing behavior in childhood is a public health priority not only due to its prevalence, but also due to its complex comorbidities and associated outcomes later in life [7]. Children with early onset disruptive behaviors tend to experience more detrimental outcomes in their adulthood including higher rates of antisocial behavior, mental health problems, and healthcare services utilization [10]. Externalizing behavior is relatively stable, resistant to treatment [7], and is more common in boys than in girls across developmental stages [11,12]. Although less common, girls with disruptive behaviors are at an increased risk for depression, self-harm, post-traumatic stress disorder, substance abuse, interpersonal aggression, early and risky sexual behavior, and lower academic achievement [10].

### Neighborhood collective efficacy

Despite the increases in the prevalence of child and adolescent mental health and behavioral disorders in the US, the role of larger structural and societal influences on externalizing behaviors is scarcely examined in the current literature despite an increased focus on examining the role of social determinants of health (SDOH) as drivers of health outcomes in recent years. Comprising of both built environment (e.g., green spaces and zoning ordinance) and social environment (e.g., neighborhood social processes) [13,14], neighborhood environment is a potent SDOH that affects health outcomes in children spanning chronic illnesses (i.e., asthma and obesity), life expectancy [15–18] and psychological health [15,19–21].

In this sense, neighborhood collective efficacy (NCE) is an essential component of the neighborhood social environment that has not always been included in existing literature. A scoping review of neighborhood effects on early child development identified that most of the studies conceptualized neighborhood effects as neighborhood poverty (or socio-economic status), describing the association between neighborhood poverty and mental health outcomes in children [22]. However, NCE--defined as “social cohesion among neighbors combined with their willingness to intervene on behalf of the common good” [23]--is seldom included in the construct of neighborhood. NCE is a structural social-interactive mechanism in which neighborhoods with high levels of collective efficacy possess high social cohesion amongst its residents who are willing to fight to protect communal facilities and access to healthcare, maintain the neighborhoods against neglect, destruction, or abandonment [22–24]. Furthermore, NCE encompasses the sense of community held by community members, which captures residents’ sense of membership, influence, fulfillment of needs, and shared emotional connection to their neighborhood [25]. NCE has shown to be significant predictor of neighborhood crime and youth delinquency [23,26]. Based on these early findings, NCE has been further applied to various child health outcomes such as childhood obesity and adolescent mental health [27,28]. A recent systematic review evaluated the interaction between individual racial/ethnic identity and neighborhood context in differentiating developmental trajectories of conduct problems, and found that 2 studies (out of 18) revealed significant effects of NCE, such that high collective efficacy was associated with a decrease in delinquency from ages 12–15 [29]. However, to our knowledge, this is one of the first systematic reviews to evaluate the existing evidence specifically linking NCE and child externalizing behaviors across developmental trajectories.

Given that NCE is a significant, yet sometimes overlooked aspect of the neighborhood environment that may have implications for externalizing behaviors in children and adolescents, the goal of this manuscript is to conduct a systematic review to examine the role that NCE plays in child and adolescent externalizing behavior outcomes to inform practice and policy decisions. The overarching research question for this review was: What is the relationship between NCE and child externalizing behaviors across developmental trajectories? Given the early literature, we hypothesized that neighborhoods with higher NCE will be associated with decreased prevalence and/or severity of child externalizing behaviors throughout children’s developmental trajectories.

## Methods

This systematic review is pre-registered via Open Science Framework (OSF) [Registration DOI: https://doi.org/10.17605/OSF.IO/U6FJD; registered March 23, 2023] and followed the Preferred Reporting Items for Systematic Reviews and Meta-Analyses (PRISMA) guidelines [30], followed by a checklist and a flow diagram.

### Study inclusion and exclusion criteria

This study followed a set of predetermined inclusion criteria. Criteria for inclusion were: 1) use of quantitative design to assess the relationship between NCE and child/adolescent behavioral outcomes; 2) publication in a peer-reviewed journal; 3) sample that included children and adolescents (defined as under 21 years of age); the 4) measurement of NCE using the measures, or a variation of the measure, created by Sampson and colleagues, which is the only validated assessment that includes social control, social cohesion/trust, [23] and 5) measurement of externalizing behaviors using a reliable and validated measure. No time or language restrictions were applied for a comprehensive search. The search was conducted in November 2024. Several exclusion criteria were applied to this study to ensure a high-level systematic review. Meeting abstracts, dissertations, pre-prints, editorials, and commentaries were excluded. Experimental studies were excluded given that they do not address the standard condition of the neighborhood and children’s externalizing behaviors in their natural state as desired.

### Search strategy

We identified manuscripts via database searches through PubMed, PsycINFO, and CINAHL. The search string is presented in a supplemental file (S1 File). Identified publications were managed using Covidence and Zotero softwares.

### Study selection and data extraction

**Fig 1:**
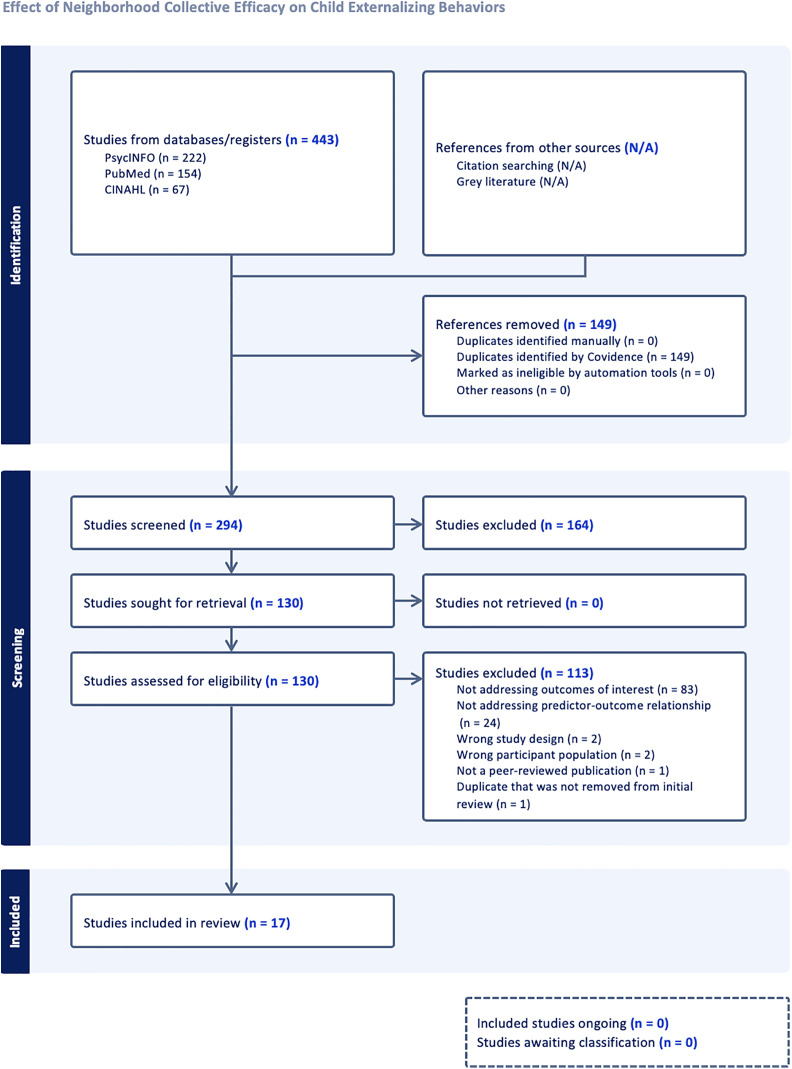
PRISMA flow chart diagram.

From employing the predetermined search strategy described above, we identified 294 manuscripts after 149 duplicates were removed. J.L. and H.P. independently screened the titles and abstracts of the manuscripts for relevancy guided by the pre-determined inclusion and exclusion criteria (Fig 1). After title and abstract review, 164 manuscripts were removed as they did not adequately answer the research question. Then, J.L. and H.S. conducted full-text reviews of the remaining 130 manuscripts with documented reasons for either inclusion or exclusion. Of the 130 manuscripts, 113 studies were excluded after full text review. All papers identified in the search and the rationale for exclusion are outlined in S1 Table. Furthermore, the reasons for exclusion are also detailed in Fig 1, which shows that the most common reason for exclusion at this stage was due to not addressing the outcome variable of interest (N = 83) and not utilizing NCE as the predictor variable and externalizing behavior as the outcome variable (N = 24). Notably, we identified one study that was published in Japanese. The full text was verified by the second author of this study who is fluent in Japanese. This study was ultimately excluded due to the age of the participants. Finally, 17 articles met the criteria for final extraction. J.L. extracted the 17 studies onto a template which included author name, publication year, purpose, sample, setting, measure of NCE and its psychometric properties, measure of externalizing behavior and its psychometrics, type of analysis, covariates, and results (Table 2). H.S. reviewed the extraction chart for accuracy and thoroughness.

**Table 1:** The National Heart, Lung, and Blood Institute (NHBLI) Quality Assessment Tool for Observational Cohort and Cross-sectional studies.

	Castillo (2020)	Ma (2016)	Ma (2017)	Ma (2018)	Pei (2022)	Wang (2020)	Pei (2024)	Hardi (2024)	Odgers (2009)	Browning (2014)	Ichikawa (2017)	Betancourt (2014)	Kofler (2022)	Moren-Cross (2006)	Emery (2015)	Liu (2016)	Sterrett-Hong (2023)
1. Was the research question or objective clearly stated?	Y	Y	Y	Y	Y	Y	Y	Y	Y	Y	Y	Y	Y	Y	Y	Y	Y
2. Was the study population clearly specified and defined?	Y	Y	Y	Y	Y	Y	Y	Y	Y	Y	Y	Y	Y	Y	Y	Y	Y
3. Was the participation rate of eligible persons at least 50%?	Y	Y	Y	Y	Y	Y	Y	Y	Y	NR	Y	NR	NR	Y	Y	NR	NR
4. Were all the subjects selected or recruited from the same or similar populations (including the same time period)? Were inclusion and exclusion criteria for being in the study prespecified and applied uniformly to all participants?	Y	Y	Y	Y	Y	Y	Y	Y	Y	Y	Y	Y	Y	Y	Y	Y	Y
5. Was a sample size justification, power description, or variance and effect estimates provided?	Y	Y	Y	Y	Y	Y	Y	Y	Y	Y	Y	Y	Y	Y	Y	Y	Y
6. For the analyses in this paper, were the exposure(s) of interest measured prior to the outcome(s) being measured?	Y	N	N	N	Y	Y	Y	Y	N	N	N	N	N	N	N	N	N
7. Was the timeframe sufficient so that one could reasonably expect to see an association between exposure and outcome if it existed?	Y	Y	Y	Y	Y	Y	Y	Y	Y	Y	Y	Y	Y	Y	Y	Y	Y
8. For exposures that can vary in amount or level, did the study examine different levels of the exposure as related to the outcome (e.g., categories of exposure, or exposure measured as continuous variable)?	Y	Y	Y	Y	Y	Y	Y	Y	Y	Y	Y	Y	Y	Y	Y	Y	Y
9. Were the exposure measures (independent variables) clearly defined, valid, reliable, and implemented consistently across all study participants?	Y	Y	Y	Y	Y	Y	Y	Y	Y	Y	Y	Y	Y	Y	Y	Y	Y
10. Was the exposure(s) assessed more than once over time?	Y	Y	Y	N	N	N	N	Y	N	N	Y	Y	N	N	N	N	N
11. Were the outcome measures (dependent variables) clearly defined, valid, reliable, and implemented consistently across all study participants?	Y	Y	Y	Y	Y	Y	Y	Y	Y	Y	Y	Y	Y	Y	Y	Y	Y
12. Were the outcome assessors blinded to the exposure status of participants?	NR	NR	NR	NR	NR	NR	NR	NR	NR	NR	NR	NR	NR	NR	NR	NR	NR
13. Was loss to follow-up after baseline 20% or less?	NR	N	N	N	NR	N	NR	N	NR	NR	N	NR	NR	NR	NR	NR	NR
14. Were key potential confounding variables measured and adjusted statistically for their impact on the relationship between exposure(s) and outcome(s)?	Y	Y	Y	Y	Y	Y	Y	Y	Y	Y	Y	Y	Y	Y	Y	Y	Y

Y= Yes, N= No, NR = Not Reported.

**Table 2:** Extraction Chart: Summary of studies included in the review (n = 17, sample size = 28,957).

Study	Purpose	Sample & Setting	Measure of Neighborhood Collective Efficacy (NCE) and Psychometrics	Measure of Externalizing Behavior and Psychometrics	Analysis and Covariates	Results
Longitudinal studies The Fragile Families and Child Wellbeing Study (FFCWS) (renamed Future of Families & Child Wellbeing Study in 2023) is a birth cohort of 4,898 families. Wave 1: Birth (1998–2000) Baseline mothers’ and fathers’ interviews conducted in-person after the focal child’s birth at hospitals Wave 2: when the child was one year old (1999–2001) (core interviews) Wave 3: 3-year-old (2001–2003) (core interviews + In-home Study + child care provider survey) Wave 4: 5-year-old (2003–2006) (core interviews + In-home Study + teacher survey) Wave 5: 9-year-old (2007–2010) (core interviews + In-home Study + child survey + teacher survey + DNA sampling) Wave 6: 15-year-old (2014–2017) (core interviews + In-home Study + child survey + DNA sampling + 5 collaborative studies)						
**1**-Castillo et al., (2020) [39]	Examine the direct and indirect relationships of four key factors on child externalizing behavior: (1) nonstandard work shift, (2) parents’ work-related stress, (3) parenting (corporal punishment and warmth), and (4) neighborhood collective efficacy (NCE)	N = 1,035 Racially diverse sample of low-income families; data collected by mothers of children at Wave 3 and Wave 4; ~ half are Black, and the other half are white and Hispanic; parents with mean income of $47,871	NCE scale by Sampson et al. Average: 3.0 (SD = 0.70); Wave 3: 2.88 (0.76); Wave 4: 3.17 (0.61) Cronbach’s α = 0.85 (Wave 3) and 0.86 (Wave 4)	CBCL externalizing behavior scale (28 items) Score at Wave 3 = 13.41 (7.49); Wave 4 = 11.27 (6.21)	One multilevel model and 7 mediation tests focal child’s sex, age, race, the mother’s marital status, parent education, and the wave of data collection	NCE was negatively associated with youth externalizing behavior (β=−0.81, *p* < .001), suggesting that as NCE increased, child externalizing behavior decreased.
**2**-Ma (2016) [40]	Explore the simultaneous role of NCE and maternal spanking on externalizing and internalizing problems in early childhood. Specifically, test the hypotheses that both the lack of NCE and maternal spanking will have positive associations with externalizing and internalizing behavior problems of 5-year-old children.	N = 2,472 Children: M = 61.11 months (SD = 2.42) and 52.1% male. Mothers: M = 30.21 years (SD = 6.01); White: 21.9%, Black 51.3%, Hispanic, 23.7%, Other 3.1%	NCE scale by Sampson et al. NCE: 3.10 (0.65) (α = 0.86) at Wave 4	CBCL/2–3, which included Aggressive Behavior subscale at Wave 3 & CBCL/4–18, which included Aggressive and Delinquent scales at wave 4; Externalizing behavior at age 3 = 0.65 (0.39)(α = 0.86) & at age 5 = 0.42 (0.25) (α = 0.86)	Multilevel modeling Controlled for maternal warmth, mother’s depression, child demographics, mother’s demographics, neighborhood demographics; Cross-sectionally examined	1. Model 1 established an inverse association between collective efficacy and externalizing behavior at age 5, after accounting for externalizing behavior at age 3 (β = −0.023 (SE = 0.007), *p* < .001). 2. In Model 2, both collective efficacy and mother’s spanking, regardless of frequency, were significant predictors of externalizing behavior, even after accounting for prior externalizing behavior score. 3. In Model 3 that included the full set of covariates, all frequencies of spanking were met with increases in externalizing behavior (β = 0.035 to 0.108, *p* = .001) at age 5 after controlling for collective efficacy and externalizing score at age 3; the effect of collective efficacy on externalizing behavior reduced both in magnitude and significance in comparison to the previous model (β=−0.013, *p* = .06).
**3**-Ma et al., (2017) [41]	Explore the associations of NCE and maternal corporal punishment with the longitudinal patterns of early externalizing and internalizing behavior problems. Specifically, (a) to examine the direct associations of NCE and maternal corporal punishment with externalizing and internalizing behavior problems in early childhood, (b) to investigate whether child age moderates the associations of NCE and maternal corporal punishment with early behavior problems, and (c) to explore whether there is an indirect effect of NCE on early behavior problems through maternal corporal punishment	N = 3,705 families Child demographics: Wave 2 = 15.02 months (SD = 3.43), Wave 2 = 35.61 months, and Wave 3 = 61.58 months & 52% male Mother’s demographics: 26.27 years (SD = 6.02) at Wave 1; 21% White, 50% Black, 26% Hispanic, 3% Other; 29% Married, 28% Cohabiting & 43% Not married or cohabiting; 34% less than high school, 31% high school degree or GED, 25% some college/technical school, 10% college degree or higher	NCE scale by Sampson et al. NCE at Wave 3: 2.91 (0.70) and at Wave 4: 3.10 (0.65); Internal consistency of the ten-item collective efficacy scale was 0.85 at Wave 3 and 0.86 at Wave 4	CBCL/2–3 (Wave 3) used Aggressive Behavior Subscale (α = 0.86); CBCL/4–18 (Wave 4) used Aggressive Behavior Subscale (α = 0.85). Externalizing Behavior at age 3 = 0.65 (0.39) & at age 5 = 0.53 (0.34)	Longitudinal multilevel models Covariates: emotional temperament, maternal warmth, mother’s depression, child and mother demographics, and neighborhood demographics	1. The longitudinal multilevel model showed that the average level of externalizing behavior significantly declined from age 3–5 (β = –0.069, *p* < .001), and NCE had a noticeable inverse association with differences in externalizing behavior at mean age, net of covariates (β = −0.040, *p* < .001). 2. Results from longitudinal multilevel models indicated that, overall, children living in neighborhoods with low collective efficacy from ages 3–5 exhibited higher behavior problems at mean age (3.1 years) of the study sample.
**4**-Ma et al., (2018) [42]	First, examine the direct associations between mother’s perception of NCE and children’s behavior and maternal corporal punishment and children’s behavior in a single model. Second, examine the moderating role of race/ethnicity in these associations.	N = 2,388 Mother’s demographics: non-Hispanic White (n = 540), non-Hispanic Black (n = 1264), or Hispanic (n = 584); respondents in the other race category in FFCWS did not yield a sufficient sample size for reliable estimates (n = 76, American Indian/Native American, Native Hawaiian/Pacific Islander, and Asian)	NCE scale by Sampson et al. NCE taken at Wave 4 core mother interview (α = 0.87); NCE: 3.10 (0.65)	CBCL Wave 3 (α = 0.86) and Wave 4 (α = 0.87) via in-home interviews Externalizing behavior at age 3 = 0.65 (0.39) & at age 5 = 0.42 (0.25)	Multilevel models with interaction terms; random intercept models Controlled for maternal warmth, mother’s depression, child demographics, mother’s demographics, neighborhood demographics Cross-sectionally analyzed	1. Perceived NCE and externalizing (r = –.14, *p* < .001) at age 5 demonstrated small but significant inverse relationship. 2. Model 1 indicates that higher values of NCE was associated with lower externalizing behavior (β = −0.01, *p* = .04). 3. Race/ethnicity did not moderate the relationship between NCE and externalizing behavior in all groups; the interaction between race/ethnicity and NCE was significant for Hispanic children’s internalizing behavior. 4. The nonsignificant interaction between NCE and race/ethnicity on externalizing behavior suggests that community-level policies and programs that aim to increase NCE may be beneficial to all children, regardless of their racial/ethnic backgrounds.
**5**-Pei et al., (2022) [43]	Aim to capture the specific pathways from neighborhood structural factors and process factors to early childhood internalizing and externalizing symptoms.	N = 2,722 children 47.43% girls, 47.6% of mothers were Black, followed by Hispanic (27.30%) and White (21.10%). Mothers’ average age was 27.58 at children’s age 5, and 68.81% of the mothers had an education less than college. A quarter (25.94%) of mothers were married to the focal child’s biological father. On average, the focal child’s household poverty ratio was 194%, and each family had approximately two children under age 18 living in the household.	NCE scale by Sampson et al. social cohesion: α = 0.88; social control: α = 0.80.	CBCL Externalizing symptoms: 12.16 (7.03), α = 0.85	Structural equation model (SEM) Child’s gender, race/ethnicity, Mother’s age, education degree, relationship with the child’s biological father, and number of children younger than 18 in the household at child’s age 3 were also controlled, and categorical variables were dummy coded	Results showed that: (a) specific types of neighborhood structural factors were directly related to either early childhood internalizing or externalizing symptoms; (b) neighborhood structural factors affected early childhood behavioral problems via both neighborhood process factors and child maltreatment experiences; and (c) only social cohesion (β = −0.11, *p* = .001), as one type of neighborhood process factors, had both direct and indirect effects on early childhood behavioral problems (social control did not have any significant effects on early childhood behavioral problems).
**6**-Wang et al., (2020) [48]	Examine the longitudinal associations between neighborhood concentrated poverty and collective efficacy, mothers’ parenting stress, exposure to adverse childhood experiences (ACEs), and later adolescent outcomes	N = 4,898 children and their mothers Mother’s demographics: 48% black, 25 years of age, 64% living in poverty; children born in unmarried families (75% unmarried) were oversampled	NCE scale by Sampson et al. Social control: 2.90 (1.38) & Social cohesion: 2.76 (1.32) Cronbach’s α = 0.88 at age 3	CBCL Behavior problems: 2.93 (1.43) Cronbach’s α = 0.91 for the entire scale; measured at age 15	Structural equation modeling with latent variables The characteristics of mothers and their children were included as covariates; child’s sex, mother’s race/ethnicity, mother’s educational attainment, marital status, cohabitation, mother’s age	1. The results suggest that NCE is associated directly and indirectly (via parenting stress and ACEs) with adolescents’ behavior problems and social skills, and indirectly with their delinquency. 2. Lower levels of NCE in early childhood were directly related to more behavior problems (β=−0.06, *p* < .05) and poorer social skills (β = 0.09, *p* < .001) of their children in adolescence. 3. Both neighborhood concentrated poverty (β = 0.06, *p* < 0.05) collectively efficacy (β=−0.26, *p* < .001) was significantly related to mothers’ parenting stress, which was significantly related positively to adolescents’ delinquency and behavior problems (β = 0.06, *p* < .05; β = 0.14, *p* < .001, respectively), and negatively to their social skills (β=−0.06, *p* < .001). 4. Lower levels of collective efficacy were associated with more ACEs (β = −0.07, *p* < .01), which were, in turn, associated with increased levels of delinquency and behavior problems (β = 0.08, *p* < .001; β = 0.15, *p* < .001, respectively).
**7**-Pei (2024) [49]	Examine the longitudinal effects of two types of early childhood neighborhood factors on the co-development of internalizing and externalizing symptoms from early childhood to adolescence	N = 2,385 children and their mothers 47.80% female; 48.60% Black, 25.40% Hispanic, and 22% White;	NCE scale by Sampson et al. collected at age 3 social cohesion: α = .88; social control: α = .80	CBCL Externalizing subscale measured at ages 3, 5, 9, and 15; Cronbach’s α = 0.85–0.89	Parallel-process growth curve modeling Controlled for the focal child’s demographic information at 3 years including gender, race, primary caregiver’s education level, poverty level, parental marital status, and number of children in the household, child maltreatment experiences, maternal drug use, and maternal mental health	Children living in neighborhoods with a higher level of social cohesion had lower initial levels of externalizing symptoms (β = −0.14, 95% CI = −0.19, −0.09, *p* < .001). A higher level of social cohesion was positively associated with changes in externalizing symptoms with a steady decrease (β = 0.06, 95% CI = 0.01, 0.12, *p* = .03).
**8**-Hardi et al., (2024) [35]	Test the cumulative and specificity effects of adversity across a range of childhood periods and contexts to prospectively predict youth internalizing and externalizing problems using a novel theory-informed data-driven approach in a diverse, longitudinal, population-based sample spanning 15 year, focusing on risk factors of parenting practices, home environment, and neighborhood environment	N = 4,210 47% females, 49% Black, 25% Hispanic, 18% White, 8% other/multiracial; $22,500 median household income at child’s birth	NCE scale by Sampson et al. Age 3 α = .84; age 5 α = .87; age 9 α = .87	Delinquency scale (13 items) adopted from the National Longitudinal Study of Adolescent Health and 5 items on youth substance use (α = .81)	Structured Life-Course Modeling Approach Controlled for ethnoracial identity, sex at birth, parental marital status, birth city, child temperament (shyness, emotionality), and pubertal development at age 15	1. Low collective efficacy at age 9 (r^2^ = 0.049%) and cumulative effect (r^2^ = 0.083%) did not significantly predict externalizing behaviors. 2. However, cumulative effect of all types of adversity (including low collective efficacy) as well as psychological aggression significantly predicted youth externalizing behaviors (r^2^ = 1.16%, *p* < .001).
E-Risk Longitudinal Twin Study is a sample drawn from a 1994–1995 birth register of twins born in England and Wales. The sample was selected in 1999–2000, when 1,116 families with same-sex 5-year-old twins participated in home-visit assessments, forming the base cohort for the study. Wave 1 (Age-5 assessment): home visit with questionnaires obtained from teachers Wave 2 (Age-7 assessment): follow up home visit with questionnaires obtained from teachers Wave 3 (Age-10 assessment): 3^rd^ follow up home visit with questionnaires obtained from teachers						
**9**-Odgers et al., (2009) [37]	Report on the influence of neighborhood-level deprivation and collective efficacy on children’s antisocial behavior between the ages of 5 and 10 years. Specifically, analysis 3 aims to answer: Does NCE predict children’s developmental course of antisocial behavior?	N = 2,232 children (1,116 families) Families drawn from the birth register were spread across England and Wales and represented the full range of socioeconomic status in Britain. All families were English speaking, and the majority (93.7%) of the families were White.	NCE scale by Sampson et al. Each of the 10 items were standardized and then averaged to create a collective efficacy total score (0.07 (0.54) ranging from −2.06 to 1.63, N = 2,176) Cronbach’s α = 0.88	CBCL delinquent and aggressive behavior scales; The Antisocial Behavior Scale was administered when the children were age 5 ((23 (17.3), α = 0.94, range = 0–130.4, N = 2,232), age 7 (20.3 (17.2), α = 0.95, range = 0–132, N = 2,178), and age 10 (19.5 (17.8), α0 = .92, range = 0–150, N = 2,138)	Latent Growth Curve Modeling Neighborhood problems, family-level factors (parental history of antisocial behavior, physical child maltreatment, domestic violence, and family socioeconomic disadvantage), and sex	1. Children in deprived versus affluent neighborhoods had higher levels of antisocial behavior at school entry (24.1 vs. 20.5, *p* < .001) and a slower rate of decline from involvement in antisocial behavior between the ages of 5 and 10 (−0.54 vs. −0.78, *p* < .01). 2. NCE was negatively associated with levels of antisocial behavior at school entry (r = −.10, *p* < .01) but only in deprived neighborhoods. 3. NCE is a robust predictor of antisocial behavior at school entry but did not predict the rate of change in antisocial behavior between the ages of 5 and 10. 4. SEM Model of neighborhood-level collective efficacy by regressing children’s level and slope antisocial behavior factors on ratings of NCE. Results from this analysis indicated that neighborhood-level collective efficacy was negatively related to children’s initial levels of antisocial behavior (y01 = −0.10, *p* < .01); children living in neighborhoods with higher levels of collective efficacy had lower levels of antisocial behavior at school entry. There was also a trend towards neighborhood-level collective efficacy predicting how quickly children’s antisocial behavior declined between the ages of 5 and 10; children in neighborhoods with higher levels of collective efficacy demonstrated a more rapid decrease in antisocial behavior across childhood.
Project on Human Development in Chicago Neighborhoods (PHDCN) Study: three-wave longitudinal cohort study with 7 cohorts of children and adolescents (over 6,000) recruited from 80 Chicago neighborhood clusters between 1995 and 1996. In-home interviews and assessments were conducted at 3 time points over a 7-year period Wave 1: 1995–1996 Wave 2: 1998–1999 Wave 3: 2001–2002						
**10**-Browning et al., (2014) [45]	1) Whether exposure to potentially lethal community violence is positively associated with both internalizing and externalizing problems among pre- and early adolescent children. 2) Test the hypothesis that neighborhood collective efficacy buffers youth against the mental health consequences of exposure to life threatening community violence (while also allowing for the possibility that collective efficacy has direct effects on mental health outcomes). 3) whether the effects of exposure to life threatening violence and any observed interactions between collective efficacy and exposure to life threatening violence varied by gender.	N = 1,227 Age 7.9–13.2 at Wave 1; Latino (50%), African American (33%), and White (14%)	NCE scale by Sampson et al. NCE score: −.01 (.99); the 3-level reliability of the combined scale was 0.63.	CBCL completed by primary caregivers; at Wave 1, the full CBCL was completed, and at Wave 2, reduced inventory was completed. Scores at Wave 2 was used as dependent variable (externalizing α = 0.89) and wave 1 was used as control. Wave 1 externalizing 10.76 (8.68); Wave 2 externalizing 7.62 (7.62)	Three-level multivariate linear models; multivariate multilevel models incorporated internalizing and externalizing scales at level one, person-level covariates at level two, and neighborhood level characteristics at level three Family background and demographic characteristics, family processes, neighborhood-level structural indicators	1. The multivariate multilevel models show that both in boys and in girls, NCE did not have main effects on externalizing behaviors; boys: (Model 1: −0.13 (0.32) & Model 2: −0.18 (0.33)); girl: (Model 1: −0.08(0.29) & Model 2: 0.17(0.27)). 2. However, in girls, collective efficacy served as a buffer against the mental health consequences of girls’ exposure to life threatening violence (Model 2: −1.79 (0.84)).
Stratification Health, Income, and Neighborhood (JSHINE) Study: a two-wave study (Wave 1 (2010) and Wave 2 (2013)) in which households recruited from Tokyo metropolitan area through cluster random sampling participated.						
**11**-Ichikawa et al., (2017) [44]	Examine the longitudinal association between change in NCE (as perceived by parents) and change in children’s psychosocial development.	N = 918 children and parents (452 households); Age range: 4–17 at Wave Sample drawn from a clustered random sample of individuals aged 25–50 years residing in 4 municipalities in urban or suburban settings of the Tokyo metropolitan area	NCE scale by Sampson et al. Informal Social Control Scale: Wave 1: 16.1 (3.6); Wave 2: 16.3 (3.5), Cronbach α = 0.87; Social Cohesion/Trust Scale: Wave 1: 16.3 (2.9); Wave 2: 16.8 (2.9), Cronbach α = 0.79	CBCL Wave 1: 50.4 (7.9); Wave 2: 49.6 (7.4)	Fixed-effects regression that controls for time-invariant observed and unobserved confounding variables. Controlled for time-varying covariates between waves, including change in family income, change of job, residential moves, change in number of family members, as well as change in the health status of family members	1. Adjusted for time-varying confounding variables, each SD increment in social cohesion remained significantly associated with a decrease in child total problem score (β = −0.22; 95% CI: −0.37 to −0.001; d = −0.03). 2. Adjusted for time-variant variables, informal social control remained significantly associated with children’s externalizing problems (β = −0.16; 95% CI: −0.30 to −0.03; d = −0.02). 3. Social cohesion was associated with a decrease in child total problem score, while informal social control was associated with lower externalizing problems, especially for boys.
**12**-Betancourt et al., (2014) [32]	Examine three community-level characteristics – social disorder and collective efficacy within the community, as reported by caregivers, and perceived stigma as reported by youth – in relation to externalizing behaviors and internalizing symptoms among male and female former child soldiers in post conflict Sierra Leone.	N = 243 Former child soldiers and their primary caregivers in Sierra Leone; 30% female, Mean age = 16.6 years	NCE scale by Sampson et al. 3.7 (0.6) in 2004 3.8 (0.6) in 2008 Scale: 0(low)-4(high)	Oxford Measure of Psychosocial Adjustment (OMPA). The externalizing subscale includes 12 items and evaluates hostile or aggressive behavior over the past 6 months. The scale demonstrated good internal consistency in this sample (α = 0.86 in 2004; α = 0.80 in 2008). Between 2004 and 2008, externalizing behaviors depreciated by approximately 33%, from an average OMPA externalizing score of 8.7 to 5.8 (scale range: 0–36)	Two-point growth models were estimated to examine the relationship between explanatory variables and mental health outcomes in 2004 (baseline) and 2008 (follow-up). Individual level covariates such as having been a victim of rape and having penetrated violence, etc. Time-varying covariates: social disorder, collective efficacy, family acceptance, and child soldier stigma	No relationship between collective efficacy and externalizing was noted (β = 0.01, *p* > .05); Overall, the final two-point growth model accounted for 43% of variance in the intercepts (externalizing scores at baseline) and 42% of the variance in slopes (change in scores over time).
Cross-sectional studies						
**13**-Kofler et al., (2022) [46]	Examine the moderating effects of resting heart rate (a measure of biological sensitivity) and sex on the relationships between NCE, psychopathic traits, and antisocial behaviors.	N = 245 8–11-year-old boys and girls and their primary caregivers (Mean age = 10.03, SD = 0.59) from Brooklyn, NY. Participating children were 47.8% males, 8.4% Hispanic, 22.4% Caucasian, 47.7% African American, 3.4% Asian, and 18.1% mixed/other	NCE scale by Sampson et al. Informal social control subscale: 15.23 (5.95) and 14.67 (6.00) for boys and girls, respectively. Cronbach’s α = 0.90 & 0.89 for boys and girls. Social cohesion subscale: 16.90 (3.50) and 16.62 (3.72) for boys and girls respectively. Cronbach’s α = 0.78 & 0.82 for boys and girls	CBCL Aggression subscale: boys: 4.71 (4.38), Cronbach’s α = 0.84 & girls: 3.73 (4.20), α = 0.84, Delinquency subscale: boys: 2.03 (2.32) Cronbach’s α = 0.71 & girls: 1.53 (1.67) α = 0.55	Hierarchical regression analyses Analyses controlled for social adversity (SA) to better isolate the effects of neighborhood social processes	1. Hierarchical regression analysis showed that there was significant 3-way interaction between social control, resting HR, and sex on aggression (part of externalizing behavior construct) (β = 0.10, *p* < .001); however, social process alone did not have a significant effect on NCE. The regression analysis showed that aggression and delinquency (which constitute externalizing behavior) were associated with social control, and the relationship was moderated by heart rate and sex.
**14**-Moren-Cross et al., (2006) [33]	Examine the relationship between maternal subjective neighborhood attributions and their children’s behavioral problems.	N = 576 former Head Start children and their mothers Child demographics: 49% male, 44% African American, 7% Hispanic, 6% Other races; Mom’s education: 1.95 (0.82), poverty distribution: 0.90 (0.64)	Mother’s perception of neighborhood characteristics included 6 domains (25 questions) which included NCE scale by Sampson et al. NCE: 20.19 (5.78) Cronbach’s α = 0.85)	Social Skills Rating System (SSRS) Problem behavior: 11.68 (5.50) Cronbach’s α = 0.85	Ordinary least squares regression Gender, race/ethnicity, father’s involvement in childcare, mother’s highest education, family income (poverty)	Neighborhoods lacking in collective efficacy are associated with children with elevated problem behavior (β = 0.094, *p* < .05). More specifically, the greater the levels of social cohesion and social control within a neighborhood, the less problem behavior.
**15**-Emery et al., (2015) [47]	Develop and introduce a measure of neighborhood ISC_CM, examine its relationship to child maltreatment, and examine its protective role in the maltreatment behavior problems relationship.	N = 293 families Most of the parents were married (90%), did not own a car (14%), and worked full time (64%). A little more than half of the respondents were mothers (53%), and the average household income was $250/month. The rate of any IPV in the last year by the father was 27.6%	NCE scale by Sampson et al. Neighborhood social control: 11.53 (2.40) Cronbach’s α = 0.77; Neighborhood solidarity: 2.06 (0.55), Cronbach’s α = 0.85	CBCL Externalizing: 45.16 (8.42) Cronbach’s α = 0.93)	Random effects logit and regression models Controlled for the age and sex of the child, as well as family size. Household income is measured in US dollars and parent’s age, length of residence and education are measured in years. Car ownership, full time employment, parent’s sex and marital status are measured as dichotomous variables.	1. The informal social control dimension of perceived collective efficacy (neighborhood control of crime in general) was associated with significantly higher levels of externalizing behavior; however, it was not significantly associated with internalizing behavior problems. 2. Found that protective ISC_CM is associated with lower odds of very severe physical abuse and lower reported externalizing problems when abuse is present. Perceived collective efficacy and punitive ISC_CM is not associated with lower odds of very severe physical abuse.
**16**-Liu et al., (2016) [34]	1) Establish the latent structure of internalizing and externalizing problems among a sample of high-risk youth (African American adolescents residing in high-poverty neighborhoods). 2) Examine the impact of multiple types of stressors on the latent factors underlying internalizing and externalizing problems. 3) Examine an uncommonly studied but potentially important protective factor, collective efficacy, to advance the understanding of neighborhood impacts on minority adolescents’ mental health.	N = 592 African American adolescents 291 male, Mage = 15.9 yrs (SD = 1.43 yrs); Recruited from Mobile Metropolitan Statistical Area in Alabama. Among the sample, 81.9% lived in a household with less than $20,000 annual income.	NCE scale by Sampson et al. NCE: 3.2 (0.80), Cronbach’s α = 0.77	Youth Self Report (YSR) Externalizing Problems T Score = 51.6 (11.43), Cronbach’s α = 0.90	1st aim: CFA; 2nd aim: SEM; 3rd aim: interaction terms between collective efficacy and the three stressor types were computed and entered to the SEM as predictors for the latent factors. Covariates varied by analysis; controlled for age, gender, and comorbid problems (stress, racial discrimination, exposure to violence, collective efficacy)	1. Collective efficacy was associated with both lower specific externalizing problems and comorbid problems. Moreover, high collective efficacy buffered the effects of stressful life events and racial discrimination on comorbid problems. 2. NCE appeared to be a distinct predictor of internalizing/externalizing problems, as it was not correlated with any of the three stressor types assessed in the study. 3. Externalizing factor was significantly inversely related to collective efficacy (β = −0.16, *p* < .05).
**17**-Sterrett-Hong et al., (2023) [50]	Examine the influence of positive and negative neighborhood conditions, in the context of genetic risk, on behavioral difficulties among low-income African American adolescents.	N = 524 (Mean age = 15.89, SD = 1.42) African American youth and their parents in high-poverty neighborhoods	NCE scale by Sampson et al. Informal social control (α = 0.85) & Social cohesion and trust (α = 0.60).	CBCL and YSR Hyperactivity and inattention – 7 items on CBCL completed by parents (α = 0.79) and YSR ADHD subscale (α = 0.73). Conduct problems – 17 item CBCL completed by parents (α = 0.87) and 15 items completed by youth via YSR (α = 0.84).	Multiple regression analyses Adolescent age, gender and 10 ancestry principal components, to correct for population stratification, were entered as covariates in all models.	1. Adolescent-reported outcomes: social cohesion was significantly related hyperactivity/inattention (β = −0.09, p < .001). Higher social cohesion showed significantly lower levels of conduct problems (β = −0.12, p < .001). Informal social control did not reveal significant relationships with youth-reported hyperactivity/inattention and conduct problems. 2. Parent-reported outcomes: Neither social cohesion nor informal social control were significant predictors for parent-reported hyperactivity/inattention and conduct problems.

* Table 2 note. Data extraction was conducted by J.L. throughout November-December 2024. All studies included in the extraction tables were deemed eligible for inclusion in the review. The extracted data includes all necessary information to replicate the analyses.

### Quality assessment

We assessed study quality using the guidelines from the National Heart, Lung, and Blood Institute (NHBLI) Quality Assessment Tool for Observational Cohort and Cross-sectional studies (Table 1) [31].

## Results

### Study characteristics

Among the 17 studies included in this review and in the extraction chart (Table 2), the sample sizes ranged from 243 to 4,898 with an average sample size of 1,738 (SD = 1,465). Most samples (N = 16) were comprised of caregiver-child (or children) dyads, with one study which included only adolescents without their caregivers. Most studies utilized participants from the US (13 out of 17) in addition to studies from the United Kingdom, Japan, Sierra Leone, and Vietnam. Fifteen out of 17 studies were published within the past 10 years (9 were published within the past 5 years, as of 2024). There was no missing data in the included studies.

Study designs included 12 longitudinal studies and 5 cross-sectional studies. All studies used Neighborhood Collective Efficacy scale created by Sampson and colleagues to quantify NCE, which is comprised of two subscales: informal social control and social cohesion/trust. The informal social control subscale includes 5 Likert-type items that ask respondents about the likelihood that their neighbors could be counted on to intervene in various ways for the greater good of the neighborhood. The social cohesion and trust subscale also includes 5 Likert-type items in which respondents are asked about how strongly they agree that the neighbors are willing to help each other out and can be trusted [23]. Most studies (13 out of 17) used the CBCL to measure externalizing behavior. The rest of the manuscripts used Oxford Measure of Psychosocial Adjustment (OMPA) [32], Social Skills Rating System (SSRS) [33], Youth Self Report (YSR) [34], and questions adopted from the National Longitudinal Study of Adolescent Health [35]. While similar with CBCL, YSL is designed to be answered by adolescents rather than their parents or caregivers [36]. Finally, it is important to note while all studies studied externalizing behavior outcomes, they used varying terms to describe the same concept (i.e., “antisocial behavior”, “delinquency”) [37].

### Data synthesis

Most of the included studies (Number (N) =15 of 17) found a significant relationship between NCE and externalizing behaviors (Table 2). We grouped key findings from selected studies, into the following themes: 1) Externalizing behavior outcomes by developmental stages (early childhood, middle childhood, and adolescence); 2) Contextual factors (moderating, mediating) that contribute to the strength and directionality of the relationship between NCE and externalizing behaviors.

1) **Developmental stages**

We define early childhood as ages 12 months to 5 years 11 months and 30 days, middle childhood as 5 years to 11 years 11 months and 30 days, and adolescence as 11 years to 20 years 11 months and 30 days. We followed the age windows created by Environment Influences on Child Health Outcomes (ECHO) Program by the National Institutes of Health (NIH) [38]. According to ECHO, the life stages intentionally overlap given that there is age-variability in normal maturation and development. If the included studies were comprised of participants who overlapped between age groups, we categorized the studies by the age group that included the majority of participants.

### Early childhood (12 months to 5 years 11 months 30 days)

In this systematic review, all 5 studies that examined the relationship between NCE and externalizing behaviors in early childhood yielded significant associations [39–43]. The included studies in this age group were each evaluated for its quality of evidence. While there some differences across the 5 studies in whether NCE was measured prior to CBCL and whether NCE was assessed more than once over time, all studies were deemed to be of high-quality evidence in their clear statement of objectives, defined study populations, and sufficient timeframe to observe an association between NCE and externalizing behaviors. Notably, all studies in this age group were derived from the Future of Families and Child Wellbeing Study (FFCWS; formerly Fragile Families), which is a prospective birth cohort of 4,898 children born between 1998 and 2000 and their parents, spanning 20 large, urban cities in the US. Specifically, the studies examined children at 3 and 5 years of age (represented by Wave 3 and 4 from the FFCWS cohort, respectively). Given the strength of the FFCWS cohort, some studies examined the relationship longitudinally. For instance, Ma and Grogan-Kaylor examined the association of NCE with longitudinal patterns of early externalizing behavior problems (N = 3,705) [41]. The longitudinal multilevel model showed that the average level of externalizing behavior significantly declined from age 3–5 (β = –0.069, *p* < .001), and NCE had a noticeable inverse association with differences in externalizing behavior at mean age, net of covariates (β = –0.04, *p* < .001); however, some studies examined the relationship between NCE and externalizing behavior cross-sectionally. For instance, Ma et al. examined direct associations between mothers’ perception of NCE and children’s behavior (N = 2,388) assessed at Wave 4 [42]. The study found that perceived NCE and externalizing behavior problems at age 5 demonstrated small but significant inverse relationship (*r* = −.14, *p* < .001). In addition, higher values of NCE were associated with lower externalizing behavior (β = −0.01, *p* = .04). Finally, Pei et al. aimed to capture the specific pathways from neighborhood structural factors and process factors to early childhood externalizing symptoms (N = 2,722) [43]. NCE was collected at Wave 3 and early childhood behavioral problems were measured at Wave 4. The analysis showed that social cohesion had both direct and indirect effects on early childhood behavioral problems (β = −0.11, *p* = .001) while social control did not have any significant effects on early childhood behavioral problems. In summary, all studies included in this age group were of high quality evidence and demonstrated significant relationship between NCE and child externalizing behaviors, both cross-sectionally and longitudinally.

### Middle childhood (5 years to 11 years 11 months and 30 days)

Six studies [33,37,44–47] focused on the relationship between NCE and externalizing behavior in middle childhood. Three of the studies in this category used longitudinal approaches [37,44,45] while the rest [33,46,47] (n = 3) employed cross-sectional approaches. The study population in this developmental stage spanned the U.K., Japan, US, and Vietnam. Most studies described significant relationship between NCE and child externalizing behavior in middle childhood, while one study did not show significant relationship [45]. The included studies in middle childhood were also each evaluated for its quality of evidence. While there some differences across the studies in the rate of participation and whether NCE was assessed more than once over time, all studies were deemed to be of high-quality evidence in their inclusion of clear statement of objectives, defined study populations, and the inclusion of key covariates.

A significant longitudinal relationship between NCE and child externalizing behavior was shown in Ichikawa et al. in which they examined the longitudinal association between change in NCE and change in children’s psychosocial development in Japanese children (Age range: 4−17 at Wave 1; N = 918) and found that social cohesion was associated with a decrease in child total problem score (β = −0.22; 95% CI: −0.37 to −0.001; d = −0.03), while informal social control was associated with lower externalizing problems (β = −0.16; 95% CI: −0.30 to −0.03; d = −0.02) [44]. A significant cross-sectional relationship between NCE and child externalizing behavior was shown in the study by Moren-Cross et al., in which the relationship between maternal subjective neighborhood attributes and child behavioral problems was examined in third grade children (N = 576) [33]. Specifically, they found that neighborhoods lacking in NCE were associated with children with elevated problem behaviors (β = 0.094, *p* < .05). Finally, Browning et al. investigated whether NCE had direct effects on mental health outcomes and whether NCE buffers children against mental health consequence of life-threatening community violence [45]. They found that NCE did not show main effects on externalizing behaviors in both boys and girls, but it functioned as a buffer against mental health consequences of girls’ exposure to life-threatening violence. Overall, the studies included in this age group were of high quality evidence and mostly demonstrated a significant relationship between NCE and externalizing behaviors in middle childhood.

### Adolescence (11 years to 20 years 11 months and 30 days)

Finally, 6 studies [32,34,35,48–50] examined the relationship between NCE and externalizing behavior in adolescence. Four studies examined the relationship longitudinally [32,35,48,49], while two did so cross-sectionally [34,50]. The adolescent population also varied greatly spanning former child soldier adolescents in Sierra Leon [32], African American adolescents in the US [34,50], and youth from the FFCWS cohort [35,48,49]. Of the 6 studies, 5 demonstrated a significant relationship between NCE and externalizing behaviors in adolescence, while one study did not show a significant relationship. The included studies in adolescence were also each evaluated for its quality of evidence. While there also differences across the studies in the rate of participation and whether NCE was measured prior to externalizing behaviors, similar to other developmental stages, all studies were deemed to be of high-quality evidence in other critical areas including clear statement of objectives, defined study populations, inclusion of key covariates, and clearly defined outcome variables that were applied consistently across all participants.

Several studies have highlighted the significant longitudinal relationship between NCE and externalizing behaviors in adolescence. Pei explored this relationship using data from the FFCWS cohort. The study examined NCE collected from caregivers in early childhood (at age 3) and externalizing behavior outcomes measured at ages 3,5,9, and 15 using parallel-process growth curve modeling, and found that children living in neighborhoods with a higher level of social cohesion had lower initial levels of externalizing symptoms (β = −0.14, 95% CI = −0.19, −0.09, *p* < .001), which was eventually positively associated with a steady decrease in externalizing symptoms into adolescence (β = 0.06, 95% CI = 0.01, 0.12, *p* = .03) [49]. Hardi and colleagues, on the other hand, aimed to test the cumulative and specificity effects of adversity across various developmental periods to predict internalizing and externalizing behaviors in youth. While low NCE at age 9 (r^2^ = 0.049%) and cumulative effect (r^2^ = 0.083%) did not significantly predict externalizing behaviors, the cumulative effect of all types of adversity (including low NCE) significantly predicted youth externalizing behaviors (r^2^ = 1.16%, *p* < .001) [35]. While both studies used data from the FFCWS cohort, how they operationalized externalizing behaviors, their sample sizes, study aims, and methodological approaches differed significantly. Despite these differences, both studies demonstrate the longitudinal relationship between NCE and externalizing behaviors in adolescence. Conversely, Betancourt and colleagues examined community-level characteristics in relation to externalizing behaviors among former child soldiers in post-conflict Sierra Leone longitudinally (N = 243; Mean age = 16.6) [32]. The study did not yield any significant relationship between NCE and externalizing behavior (β = 0.01, *p* > .05).

Finally, Sterrett-Hong and colleagues examined the influence of neighborhood conditions on behavioral difficulties among low-income African American adolescents cross-sectionally (N = 524, Mean age = 15.89, SD = 1.42). Notably, this was the only study to use both adolescent-reported and parent-reported outcomes, and found that social cohesion was significantly related to adolescent-reported hyperactivity and inattention (β = −0.09, p < .001), and higher social cohesion showed significantly lower levels of adolescent-reported conduct problems (β = −0.12, p < .001) [50]. Interestingly, the same study found that parent-reported measure of NCE was not a significant predictor for hyperactivity/inattention and conduct problems in adolescence. In summary, the studies included in this age group were of high quality evidence, and mostly revealed a significant relationship between NCE and externalizing behaviors in adolescence.

2) **Contextual factors**

There were several contextual factors highlighted in the studies pertaining to the relationship between NCE and externalizing behavior, including corporal punishment, race and ethnicity, adverse childhood experiences (ACEs), and parenting stress.

### Corporal punishment

There were 4 studies that examined the effect of corporal punishment in the context of NCE and externalizing behaviors [39–42]. Castillo and colleagues examined whether the relationship between NCE and child externalizing behavior was mediated by mothers’ use of corporal punishment and found a significantly mediated relationship (β = −0.11, *p* < .01) [37]. Ma and Klein found that, in a random intercept model exploring simultaneous effects of perceived NCE and maternal corporal punishment on externalizing behaviors on 5-year-olds, higher NCE was associated with lower externalizing behaviors (β = −0.01, *p* = .040) and increased frequency of corporal punishment predicted higher levels of externalizing behaviors [42]. On the contrary, 2 studies found was that maternal spanking was positively associated with behavior problems but did not find that the associations of low NCE with externalizing problems were mediated by maternal spanking [40,41]. While these studies demonstrated the direct effect of corporal punishment on child externalizing behavior problems, we found limited evidence for the mediating effect between NCE and behavior problems in children.

### Race and ethnicity

Ma and colleagues examined whether race and ethnicity moderate the association between neighborhood and early behavior problems [42]. They found that Black and Hispanic mothers reported lower levels of NCE compared to their White counterparts. They also found that perceived NCE and externalizing problems at age 5 demonstrated small but significant inverse relationships. To examine the moderating effect of race/ethnicity on this relationship, they examined the simple NCE slopes for each racial and ethnic groups. Results indicated that race and ethnicity (categorized as White, Black, and Hispanic) did not moderate the association between NCE and externalizing behavior in this study.

### Adverse childhood experiences (ACEs) and parenting stress

Finally, Wang and colleagues examined the longitudinal associations between NCE and later adolescent outcomes [48]. Specifically, they examined if ACEs and parenting stress mediate the association. The study found that NCE was significantly related to mothers’ parenting stress (β = −0.26, *p* < .001), which was positively related to adolescent’s delinquency and behavior problems (β = 0.06, *p* < .05; β = 0.14, *p* < .001, respectively). In addition, lower levels of NCE were associated with more ACEs (β = −0.07, *p* < .01), which were associated with increased levels of delinquency and behavioral problems (β = 0.08, *p* < 0.001; β = 0.15, *p* < .001, respectively).

## Discussion

This systematic review examined the relationship between NCE and child externalizing behaviors spanning early childhood to adolescence. Seventeen studies with a total of n = 28,957 caregiver-child (or children) dyads and 592 adolescents were included in the review. Consistent with previous literature highlighting the importance of neighborhood condition on child/youth mental health outcomes [28,51], this systematic review yielded significant relationship between NCE and child externalizing behaviors across diverse populations of children and adolescents and their families. Specifically, this review echoed some of the existing systematic reviews that have examined other neighborhood characteristics and externalizing behaviors in children. For instance, Brumley and Jaffee (2016) systematically reviewed factors that decrease likelihood of externalizing behaviors in children and adolescents and found that both positive neighborhood effects (i.e., good housing quality) and the absence of negative neighborhood factors (i.e., exposure to marijuana in the neighborhood) were both associated with lower externalizing behaviors in children [52]. Furthermore, Jennings and colleagues’ (2018) systematic review demonstrated that there were robust linkages between various neighborhood factors (including neighborhood crime and neighborhood adversity) and externalizing behaviors in children [53]. Our systematic review builds on these previous works by demonstrating that, in addition to other neighborhood factors, NCE shows significant effect on child externalizing behaviors across the developmental trajectory. In addition, our study found that the most robust and consistent evidence for the association between NCE and externalizing behavior in early childhood compared to older developmental periods, in which all the evidence found in early childhood yielded significant relationship between NCE and externalizing behaviors. This finding is aligned with previous literature which demonstrates that externalizing behavior is manifested differently across developmental trajectories. For instance, young children may exhibit more physical aggression while adolescents may engage in more socially delinquent behaviors [54]. In addition, aggression and opposition, which are behavior clusters in externalizing behavior, tend to decrease in adolescence compared to early childhood [55]. Overall, this finding suggests that early childhood may be an important developmental window in which children may be more susceptible to neighborhood-level influences rather than in older children, who may be less susceptible to the same influences due to a variety of reasons including more time spent in other social settings (i.e., school) [56]. Additionally, neighbors may generally be more attentive to caring for and protecting younger children compared to adolescents, as adolescents with mental health issues or placed in out-of-home environments (i.e., group homes, foster care, or institutional care) often report experiencing stigmatization [57,58].

While most studies resulted in significant relationship between NCE and child externalizing outcomes both longitudinally and cross-sectionally, several studies resulted in mixed findings. Interestingly, the studies that resulted in mixed or non-significant findings were more likely to be from countries outside of the US. For instance, Odgers and colleagues’ study, based in the U.K., found that NCE was negatively associated with levels of antisocial behavior at school entry but only in deprived neighborhoods [37]. Betancourt and colleagues’ study, based in Sierra Leone, did not find significant relationship between NCE and externalizing behaviors [32]. Finally, Emery and colleagues’ study, based in Vietnam, found that only one of the constructs of NCE, informal social control, was associated with significantly higher levels of externalizing behavior [47]. While it is difficult to draw concrete conclusions, we suspect several reasons for this interesting finding: 1) Developed and widely applied in the US, the measure of NCE may have limited external validity outside of the US (i.e., countries outside of the US may conceptualize the construct of neighborhood differently); 2) Neighborhood level disparities (and other types of disparities) may be more pronounced in the US compared to other countries, leading to more significant findings; 3) Literature shows that the burdens of child and adolescent mental and behavioral health disorders are more pronounced in North American, European, and Australian countries compared to their Asian or African counterparts [59] 4) Researchers in the US may have stronger biases towards over-sampling individuals from disadvantaged and minority groups (e.g., FFCWS cohort) [39].

Finally, we found limited evidence for mediating effects of corporal punishment, parenting, and adverse childhood experiences between NCE and child externalizing behaviors, which illustrate the complex nature in which NCE influences child externalizing behaviors. Of all the mediating variables examined in this review, corporal punishment was the most frequently examined. Previous literature has shown that the use of corporal punishment predicts child externalizing behaviors [60]. However, larger societal contexts in which corporal punishment occurs is less frequently examined. In our systematic review, one of the four studies showed that use of corporal punishment mediates the relationship between NCE and externalizing behavior, while others did not. The mixed finding may be because other types of neighborhood social processes--including neighborhood violence--may more strongly predict the use of corporal punishment because the more parents perceive their neighborhood as violent, the more likely they are to engage in use of aggressive discipline [61]. While neighborhood violence and NCE are related (i.e., neighbors show less social cohesion and trust if the neighborhood deemed violent) [62], NCE may not be the most robust neighborhood-level predictor when examining corporal punishment as the mediator for child externalizing behavior outcomes.

### Clinical implications

There are many persistent challenges in addressing the current childhood mental and behavioral health crisis in the US. For one, shortage of child psychiatrists in the US is well documented [63]. This is especially concerning given that the shortage tends to be concentrated in rural and high-poverty areas in the US. Adding to the concern, Hoffmann and colleagues found that areas with mental health workforce shortages were associated with an increased youth suicide rate [63]. Given the demonstrated mental health workforce shortage and other related barriers, mental health providers may not have the means and the resources to address how neighborhood-level barriers, such as NCE, may be play a role in child behavioral health concerns. However, given the growing evidence that structural level characteristics affect child behavioral health outcomes, child mental and behavioral health providers should consider these as potential targets for interventions. Indeed, there has been increasing recognition amongst clinicians to focus on primary prevention of pediatric mental health crisis by engaging community stakeholders including the school systems and community mental health therapists [64].

In addition to the issues related to the mental health workforce, there has been limited evidence for interventions developed and tested to improve neighborhood collective efficacy in disadvantaged communities. One example of such intervention was conducted by Ohmer (2016) in which a pilot community-based intervention to facilitate collective efficacy among youth and adult residents was administered in the southeastern United States. The intervention included community organizing and mobilization, a training program to facilitate collective efficacy, and a community-based project developed by participants to address a youth violence prevention issue, and it resulted in increased levels of collective efficacy reported by the participants [65]. Building on the promising effects of interventions targeting NCE in disadvantaged communities, we recommend fostering collaborations among clinicians, policymakers, researchers, and community representatives. Such partnerships are essential for adapting and scaling these interventions to better address the behavioral and mental health needs of children and youth in the community.

Finally, this systematic review revealed the strongest evidence between NCE and child externalizing behaviors in early childhood. Therefore, researchers, clinicians, and child mental health workers should focus on this important early developmental period as a window of opportunity to find solutions to close the gap on health inequities later in life [56].

### Limitations

Due to limited number of studies to date examining the relationship between NCE and child externalizing behavior outcomes, this systematic review included only 17 studies, 5 of which employed cross-sectional approaches. Furthermore, amongst the longitudinal studies (12 studies), 2 of them examined the variables of interest cross-sectionally. The heterogeneity of the study designs and the diverse measures used to operationalize externalizing behaviors prevented the possibility of conducting meta-analysis.

While most studies utilized CBCL to measure the degree of externalizing behaviors, 4 studies used different measures (YSR, SSRS, OMPA, and questions adopted from the National Longitudinal Study of Adolescent Health) which could have affected the outcome analyses given the inherent differences in the measures in quantifying externalizing behaviors.

Of the included studies, 8 employed samples from the FFCWS cohort, which includes racially diverse sample of low-income families, with racial and ethnic minorities and unmarried mothers being overrepresented [64]. It should be noted that the overlap in the study population could have been a possible source of bias in the study outcome.

Additionally, internalizing and externalizing behaviors are typically correlated with one another, and thus, can make it challenging to draw the conclusions that NCE is associated only with externalizing behavior outcomes without controlling for the associations with internalizing behaviors [66].

The systematic review included studies published throughout the world, but most studies (n = 13) resulted from the US. Literature shows that compared to other high-income countries, the US represents one of the highest geographic health disparities alongside one of the worst other health-related outcomes and inequalities in developed nations [67,68]. Given that there was scant representation of other continents and countries in this systematic review, there could have been more pronounced bias towards finding significant relationship between NCE and externalizing behaviors given the over-representation of participants from the US. Given these limitations, the findings may not be as generalizable outside of the US.

## Conclusions

This is one of the first systematic review to examine the relationship between NCE and externalizing behaviors in children and adolescents. Notwithstanding the limitations, we found significant inverse relationship between NCE and child externalizing behaviors both cross-sectionally and longitudinally in this review, with the strongest evidence found in early childhood compared to later developmental periods. Furthermore, we found limited evidence of the mediating role of corporal punishment, parenting, and ACEs between NCE and child externalizing behaviors, suggesting areas for future research. Finally, we found that the relationship between NCE and externalizing behaviors was more pronounced in US-based studies compared to other countries. Future investigations should further examine underlying mechanisms of this relationship. This review demonstrates that structural neighborhood-level influences, especially neighborhood social processes, indeed play a critical role in child mental and behavioral health disorders.

## Supporting information

S1 File Search Strategy. The following search strategy was applied to the three databases:PubMed, PsychINFO, and CINAHL.
(DOCX)

S2 File PRISMA Checklist. (DOCX)

S1 Table Tables of all studies identified and screened and the rationale for exclusion if applicable. S1 Table 1 details studies that were excluded during title and abstract screening and the rationale for exclusion (N = 164). S1 Table 2 details studies in which the full texts were examined and were either excluded (N = 113) or included (N = 17) (Total N = 130). All identified studies in the literature search and rationale for exclusion if applicable were described through both S1 Table 1 and S1 Table 2 (N = 294).
(DOCX)
