# Supplementary material for: Neighborhood collective efficacy and children and adolescents’ externalizing behaviors across development: A systematic review
Source: PLoS One. 2026 Jan 23;21(1):e0337512. doi: 10.1371/journal.pone.0337512 (PMC12829874; doi:10.1371/journal.pone.0337512)
Supplement: S1 File — The following search strategy was applied to the three databases:PubMed, PsychINFO, and CINAHL. (DOCX) [file pone.0337512.s001.docx]

**Supplementary materials**

**S1 File. Search Strategy.** The following search strategy was applied to the three databases: PubMed, PsychINFO, and CINAHL.

(Neighborhood) AND (collective efficacy) AND (child behavior OR externalizing OR behavioral OR psych* OR develop* OR behavior* OR behavioral dysregulation OR dysregulation OR temperament OR conduct) AND (child* OR toddler OR infant OR pediatric OR adolescent OR adolescence OR pre-adolescent OR pre-adolescence OR teen)
