# Supplementary figures and images for: Neighborhood collective efficacy and children and adolescents’ externalizing behaviors across development: A systematic review
Source: PLoS One. 2026 Jan 23;21(1):e0337512. doi: 10.1371/journal.pone.0337512 (PMC12829874; doi:10.1371/journal.pone.0337512)

**Supplementary materials**

**S2 File. PRISMA Checklist.**


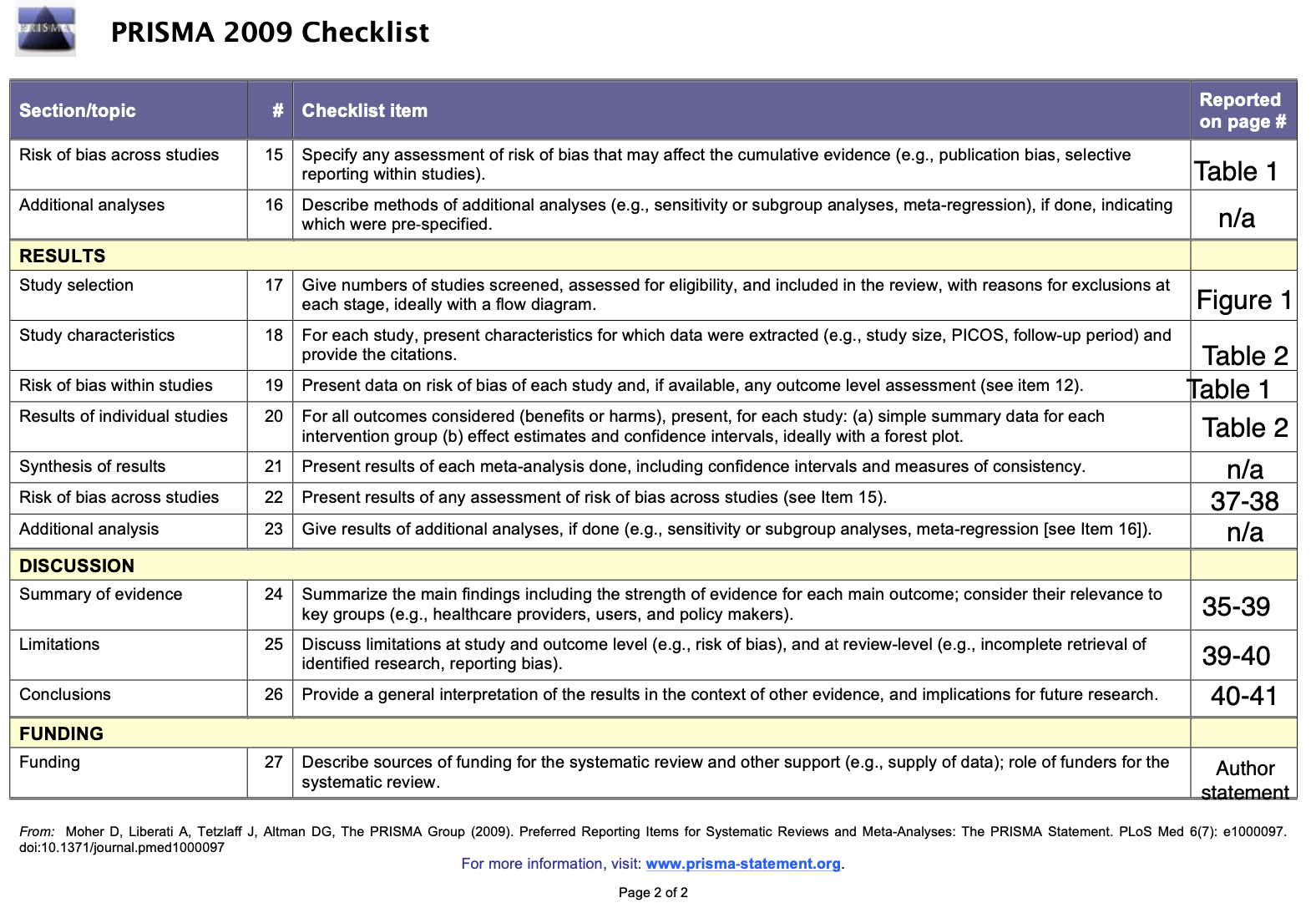


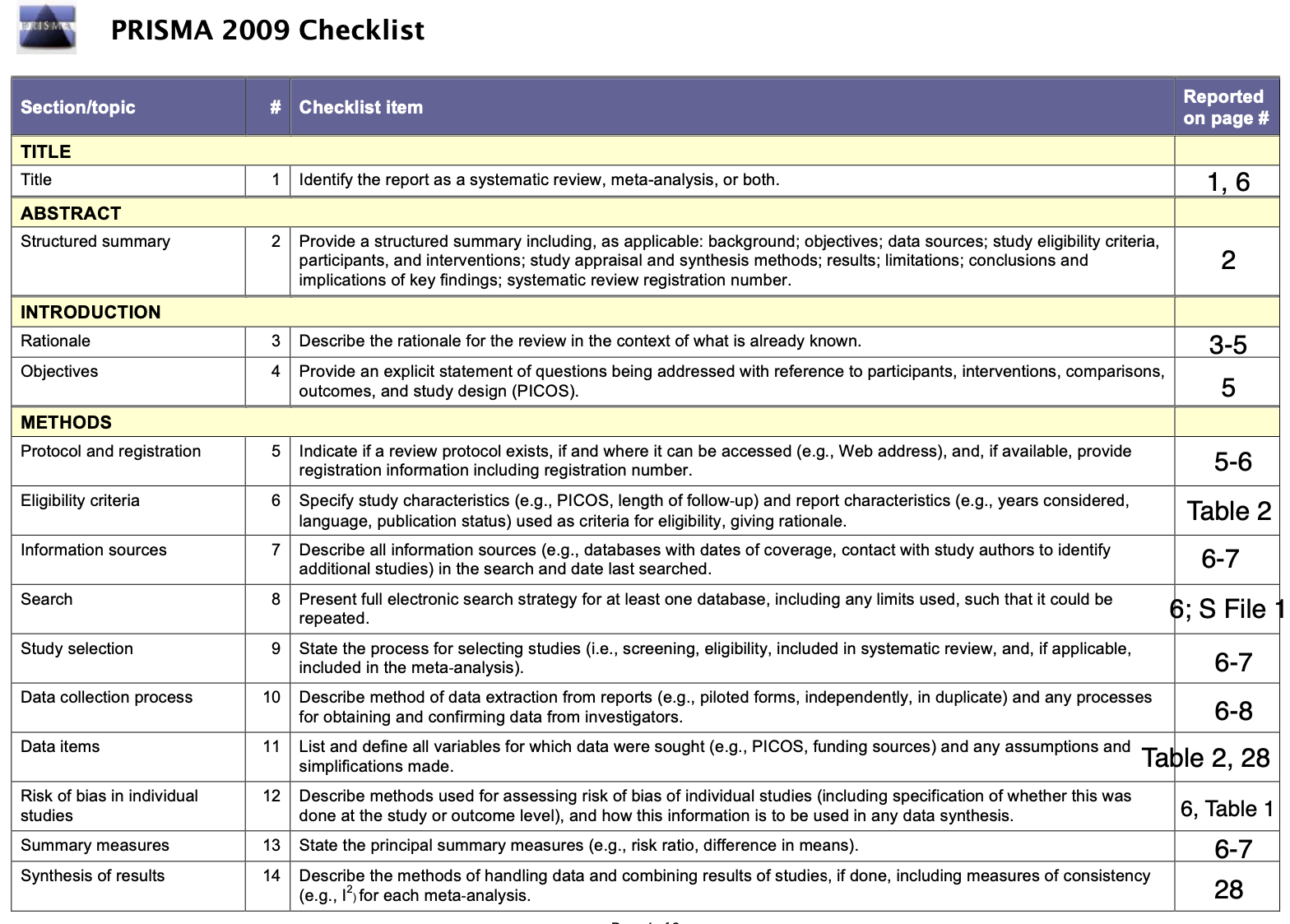

Supplement: S2 File — (DOCX) [file pone.0337512.s002.docx]
