# Supplementary material for: Neighborhood collective efficacy and children and adolescents’ externalizing behaviors across development: A systematic review
Source: PLoS One. 2026 Jan 23;21(1):e0337512. doi: 10.1371/journal.pone.0337512 (PMC12829874; doi:10.1371/journal.pone.0337512)
Supplement: S1 Table — 1 details studies that were excluded during title and abstract screening and the rationale for exclusion (N = 164). S1 Table 2 details studies in which the full texts were examined and were either excluded (N = 113) or included (N = 17) (Total N = 130). All identified studies in the literature search and rationale for exclusion if applicable were described through both S1 Table 1 and S1 Table 2 (N = 294). (DOCX) [file pone.0337512.s003.docx]

**Supplementary materials**

**S1 Table. Tables of all studies identified and screened and the rationale for exclusion if applicable.** S1 Table 1 details studies that were excluded during title and abstract screening and the rationale for exclusion (N=164). S1 Table 2 details studies in which the full texts were examined and were either excluded (N=113) or included (N=17) (Total N=130). All identified studies in the literature search and rationale for exclusion if applicable were described through both S1 Table 1 and S1 Table 2 (N=294).

S1 Table 1. Studies excluded during title and abstract screening and the rationale for their exclusion (N=164)

|  | Authors (Date) | Title of the study | Reason for Exclusion |
| --- | --- | --- | --- |
| 1. | Abdullah (2020) | Neighbourhood collective efficacy and protective effects on child maltreatment: A systematic literature review. | No child externalizing outcomes addressed |
| 2. | Ahern (2011) | Collective efficacy and major depression in urban neighborhoods. | Not focused on child-oriented outcomes; No child externalizing outcomes addressed |
| 3. | Ahern (2009) | Neighborhood smoking norms modify the relation between collective efficacy and smoking behavior. | Not focused on child-oriented outcomes; No child externalizing outcomes addressed |
| 4. | Ahern (2013) | Navigating non-positivity in neighbourhood studies: An analysis of collective efficacy and violence | Not focused on child-oriented outcomes |
| 5. | Amrhein (2017) | An analysis of outcomes in maltreated youth: The transmission of neighborhood risk through caregiver aggression and depression | Dissertation |
| 6. | Anderson (2018) | Organized Activity Involvement among Urban Youth: Understanding Family- and Neighborhood- Level Characteristics as Predictors of Involvement. | No child externalizing outcomes addressed |
| 7. | Anderson (2017) | Organized activity involvement among urban youth: Understanding predictors and mechanisms | Dissertation |
| 8. | Bamishigbin (2017) | Risk, resilience, and depressive symptoms in low-income African American fathers. | Not focused on child-oriented outcomes; No child externalizing outcomes addressed |
| 9. | Bobashev (1998) | Clusters of marijuana use in the United States. | No child externalizing outcomes addressed |
| 10. | Brisson (2014) | Neighborhoods and mental health trajectories of low‐income mothers | Not focused on child-oriented outcomes |
| 11. | Browning (2008) | Neighborhood structural inequality, collective efficacy, and sexual risk behavior among urban youth. | No child externalizing outcomes addressed |
| 12. | Browning (2013) | The Social Ecology of Public Space: Active Streets and Violent Crime in Urban Neighborhoods. | No child externalizing outcomes addressed |
| 13. | Browning (2013) | "Feeling disorder" as a comparative and contingent process: gender, neighborhood conditions, and adolescent mental health. | No child externalizing outcomes addressed |
| 14. | Burchinal (2008) | Neighborhood characteristics and child care type and quality | No child externalizing outcomes addressed |
| 15. | Burdette (2006) | Neighborhood safety, collective efficacy, and obesity in women with young children. | Not focused on child-oriented outcomes; No child externalizing outcomes addressed |
| 16. | Burt (2013) | A longitudinal examination of the relationships among disadvantaged neighborhoods, supervision, peer associations, and patterns of ethnic minority adolescent substance use | Dissertation |
| 17. | Button (2008) | Social disadvantage and family violence: Neighborhood effects on attitudes about intimate partner violence and corporal punishment | No child externalizing outcomes addressed |
| 18. | Carlson (2012) | Enhancing adolescent self-efficacy and collective efficacy through public engagement around HIV/AIDS competence: a multilevel, cluster randomized-controlled trial. | No child externalizing outcomes addressed |
| 19. | Carmona (2012) | Thriving orientation and psychological distress in youth exposed to neighborhood disorder | Dissertation |
| 20. | Caughy (2005) | Neighborhood Correlates of Cultural Differences in Perceived Effectiveness of Parental Disciplinary Tactics | No child externalizing outcomes addressed |
| 21. | Chang (2015) | Examining the direct, indirect, and moderated effects of neighborhood characteristics on trajectories of dating violence perpetration | Dissertation |
| 22. | Chen (2011) | How do neighborhoods matter across the life span? | Not a peer-reviewed research publication |
| 23. | Chen (2015) | A Comparison of the Number of Men Who Have Sex with Men among Rural-To-Urban Migrants with Non-Migrant Rural and Urban Residents in Wuhan, China: A GIS/GPS-Assisted Random Sample Survey Study. | Not focused on child-oriented outcomes |
| 24. | Child (2020) | Socioeconomic Differences in Access to Neighborhood and Network Social Capital and Associations With Body Mass Index Among Black Americans. | Not focused on child-oriented outcomes; No child externalizing outcomes addressed |
| 25. | Chou (2012) | Perceived discrimination and depression among new migrants to Hong Kong: the moderating role of social support and neighborhood collective efficacy. | Not focused on child-oriented outcomes |
| 26. | Cohen (2003) | Why is poverty unhealthy? Social and physical mediators. | Not focused on child-oriented outcomes |
| 27. | Cohen (2006) | Collective efficacy and obesity: the potential influence of social factors on health. | No child externalizing outcomes addressed |
| 28. | Conlon (2009) | Does where you live affect how you feel? An investigation into neighborhood characteristics and mental health | Dissertation |
| 29. | Connolly (2016) | Examining gene-environment interactions between antisocial behavior, neighborhood disadvantage, and collective efficacy | Dissertation |
| 30. | Coyle (2004) | Effect of health determinants on immunization rates of two-year-old children in Denton County, Texas. | No child externalizing outcomes addressed |
| 31. | Cristello (2020) | A preliminary validation of the Adolescent E-Cigarette Consequences Questionnaire | No child externalizing outcomes addressed |
| 32. | Cruwys (2020) | The recovery model in chronic mental health: A community-based investigation of social identity processes. | No child externalizing outcomes addressed |
| 33. | Dahl (2010) | In the eye of the beholder: Mothers' perceptions of poor neighborhoods as places to raise children | No child externalizing outcomes addressed |
| 34. | Daoud (2020) | Residential segregation, neighborhood violence and disorder, and inequalities in anxiety among Jewish and Palestinian-Arab perinatal women in Israel. | Not focused on child-oriented outcomes |
| 35. | Daoud (2017) | Disorganization Theory, Neighborhood Social Capital, and Ethnic Inequalities in Intimate Partner Violence between Arab and Jewish Women Citizens of Israel. | No child externalizing outcomes addressed |
| 36. | Daro (2009) | Creating community responsibility for child protection: possibilities and challenges. | Not a peer-reviewed research publication |
| 37. | Darr (2018) | Appreciating adaptive leadership in extreme context: A case study of what is working in the United Methodist Church's Chicago urban strategy | Dissertation |
| 38. | Davidson Arad (2020) | Perceived Collective Efficacy and Parenting Competence: The Roles of Quality of Life and Hope. | No child externalizing outcomes addressed |
| 39. | Davis (2021) | The interplay of community and family risk and protective factors on adjustment in young adult immigrants. | Not focused on child-oriented outcomes |
| 40. | Dawson (2019) | Parental-perceived neighborhood characteristics and adolescent depressive symptoms: A multilevel moderation analysis. | No child externalizing outcomes addressed |
| 41. | DeMarco (2009) | Welcome to the neighborhood: Does where you live affect the use of nutrition, health, and welfare programs? | No child externalizing outcomes addressed |
| 42. | Decker (2018) | Neighborhoods matter. A systematic review of neighborhood characteristics and adolescent reproductive health outcomes. | No child externalizing outcomes addressed |
| 43. | Deng (2006) | Family Processes Mediating the Relationship of Neighborhood Disadvantage to Early Adolescent Internalizing Problems | No child externalizing outcomes addressed |
| 44. | Dlugonski (2015) | Increasing collective efficacy for physical activity: Design and rationale of Moms UNITE for Health. | No child externalizing outcomes addressed |
| 45. | Domoff (2012) | College students’ perceptions of collective efficacy: Results from a nonurban sample | No child externalizing outcomes addressed |
| 46. | Drakulich (2010) | Estimating risk and expressing fear: Reactions to crime in Seattle neighborhoods | Dissertation |
| 47. | Duncan (2003) | A multilevel contextual model of neighborhood collective efficacy. | No child externalizing outcomes addressed |
| 48. | Dupéré (2012) | Neighborhood processes, self-efficacy, and adolescent mental health. | No child externalizing outcomes addressed |
| 49. | Edwards (2014) | Community matters: intimate partner violence among rural young adults. | No child externalizing outcomes addressed |
| 50. | Elliott (2017) | Youth-serving organizations and contextual moderators of associations with adolescents' antisocial behavior trajectories | Dissertation |
| 51. | Emery (2010) | Intimate partner violence relationship dissolution among couples with children: The counterintuitive role of 'law and order' neighborhoods | Not focused on child-oriented outcomes |
| 52. | Emery (2011) | Desistance from intimate partner violence: the role of legal cynicism, collective efficacy, and social disorganization in Chicago neighborhoods. | No child externalizing outcomes addressed |
| 53. | Farewell (2021) | Protective resources that promote wellbeing among New Zealand moms with young children facing socioeconomic disadvantage. | No child externalizing outcomes addressed |
| 54. | Fauth (2008) | Seven years later: effects of a neighborhood mobility program on poor Black and Latino adults' well-being. | Not focused on child-oriented outcomes |
| 55. | Fitzgerald (2008) | Assessing parenting from an ecological perspective in a Canadian city | Dissertation |
| 56. | Fleckman (2019) | Perceived social norms in the neighborhood context: The role of perceived collective efficacy in moderating the relation between perceived injunctive norms and use of corporal punishment | No child externalizing outcomes addressed |
| 57. | Fleckman (2020) | Exploring socio-ecological supports to promote healthy child development and reduce risk for child physical abuse | Dissertation |
| 58. | Fleury (2012) | Determinants associated with the utilization of primary and specialized mental health services | Not focused on child-oriented outcomes |
| 59. | Foster-Fishmasn (2009) | Who participates and why: Building a process model of citizen participation | Not focused on child-oriented outcomes |
| 60. | Franzini (2008) | Self-rated health and trust in low-income Mexican-origin individuals in Texas. | Not focused on child-oriented outcomes |
| 61. | Franzini (2005) | Neighborhood economic conditions, social processes, and self-rated health in low-income neighborhoods in Texas: a multilevel latent variables model. | Not focused on child-oriented outcomes |
| 62. | Freisthler (2015) | Understanding the interplay between neighborhood structural factors, social processes, and alcohol outlets on child physical abuse | No child externalizing outcomes addressed |
| 63. | Friedline (2017) | Do Community Characteristics Relate to Young Adult College Students' Credit Card Debt? The Hypothesized Role of Collective Institutional Efficacy. | No child externalizing outcomes addressed |
| 64. | Fromm (2004) | The processes that moderate the effect of community structural factors on neighborhood child maltreatment rates | Dissertation |
| 65. | Gaines (2012) | 'For red, for blue, for blow': Acquisition of gang membership among early adolescents | Dissertation |
| 66. | Galinsky (2012) | Is collective efficacy age graded? The development and evaluation of a new measure of collective efficacy for older adults. | Not focused on child-oriented outcomes |
| 67. | Garnett (2015) | Challenges of Data Dissemination Efforts Within a Community-Based Participatory Project About Persistent Racial Disparities in Excess Weight. | Not focused on child-oriented outcomes |
| 68. | Godbolt (2018) | Maternal fear for sons and daughters and its effect on children's outdoor activity | Dissertation |
| 69. | Gross-Manos (2019) | Two sides of the same neighborhood? Multilevel analysis of residents' and child-welfare workers' perspectives on neighborhood social disorder and collective efficacy. | No child externalizing outcomes addressed |
| 70. | Halbert (2014) | Collective efficacy and obesity-related health behaviors in a community sample of African Americans | Not focused on child-oriented outcomes; No child externalizing outcomes addressed |
| 71. | Hofer (2020) | An examination of the influence of procedurally just strategies on legal cynicism among urban youth experiencing police contact. | No child externalizing outcomes addressed |
| 72. | Humensky (2009) | The relationship between mental health and ses | Dissertation |
| 73. | Jain (2010) | Neighborhood predictors of dating violence victimization and perpetration in young adulthood: a multilevel study. | No child externalizing outcomes addressed |
| 74. | Jarrett (2011) | "Holler, run, be loud:" strategies for promoting child physical activity in a low-income, African American neighborhood. | No child externalizing outcomes addressed |
| 75. | Jensen (2018) | A typological examination of stepfamily relationship quality and adolescents' short-term and long-term adjustment | Dissertation |
| 76. | Johnson-Motoyama (2018) | Unraveling Disparities in Child Neglect Risk between Hispanics who are Immigrants and those Born in the United States: A Social-Ecological Approach Using Structural Equation Modeling. | No child externalizing outcomes addressed |
| 77. | Johnson (2015) | Neighborhood Factors and Dating Violence Among Youth: A Systematic Review. | No child externalizing outcomes addressed |
| 78. | Johnson (2016) | Father–son communication: An intervention strategy for boys and men of color to promote neighborhood safety post-Ferguson | No child externalizing outcomes addressed |
| 79. | Kepper (2016) | Parental Perceptions of the Social Environment Are Inversely Related to Constraint of Adolescents' Neighborhood Physical Activity. | No child externalizing outcomes addressed |
| 80. | Kepper (2017) | Parental perceptions of collective efficacy are inversely related to constraint of adolescents' outdoor physical activity | Dissertation |
| 81. | Kim (2015) | Community interaction and child maltreatment. | No child externalizing outcomes addressed |
| 82. | Kimbro (2011) | Neighborhood poverty and maternal fears of children's outdoor play | No child externalizing outcomes addressed |
| 83. | Kimbro (2011) | Young children in urban areas: links among neighborhood characteristics, weight status, outdoor play, and television watching. | No child externalizing outcomes addressed |
| 84. | Kimoto (2016) | Exploring the relationship between violence exposure and health risk behaviors among African American adolescent girls: A multilevel perspective | Dissertation |
| 85. | Kingston (2013) | Economic adversity and depressive symptoms in mothers: Do marital status and perceived social support matter? | Not focused on child-oriented outcomes |
| 86. | Kirk (2008) | The neighborhood context of racial and ethnic disparities in arrest. | No child externalizing outcomes addressed |
| 87. | Kristjansson (2022) | Risk and Resilience Pathways, Community Adversity, Decision-making, and Alcohol Use Among Appalachian Adolescents: Protocol for the Longitudinal Young Mountaineer Health Study Cohort. | No child externalizing outcomes addressed |
| 88. | Larouche (2019) | Relationships Between Outdoor Time, Physical Activity, Sedentary Time, and Body Mass Index in Children: A 12-Country Study. | No child externalizing outcomes addressed |
| 89. | Leddy (2019) | Community collective efficacy is associated with reduced physical intimate partner violence (IPV) incidence in the rural province of Mpumalanga, South Africa: findings from HPTN 068. | No child externalizing outcomes addressed |
| 90. | Lei (2018) | Biological embedding of neighborhood disadvantage and collective efficacy: Influences on chronic illness via accelerated cardiometabolic age. | No child externalizing outcomes addressed |
| 91. | Lenzi (2013) | How neighborhood structural and institutional features can shape neighborhood social connectedness: a multilevel study of adolescent perceptions. | No child externalizing outcomes addressed |
| 92. | Leslie (2015) | Collective efficacy, alcohol outlet density, and young men's alcohol use in rural South Africa. | No child externalizing outcomes addressed |
| 93. | Leventhal (2004) | Diversity in developmental trajectories across adolescence: Neighborhood influences | No child externalizing outcomes addressed |
| 94. | Leventhal (2009) | Neighborhood influences on adolescent development | Not a peer-reviewed research publication |
| 95. | Levy (2019) | The varying effects of neighborhood disadvantage on college graduation: Moderating and mediating mechanisms | No child externalizing outcomes addressed |
| 96. | Leykin (2013) | Conjoint Community Resiliency Assessment Measure-28/10 items (CCRAM28 and CCRAM10): A self-report tool for assessing community resilience. | Not focused on child-oriented outcomes |
| 97. | Lloyd (2019) | Neighborhood variation in the rate of child welfare contact | Dissertation |
| 98. | Ma (2016) | The effects of neighborhood disorganization and maternal corporal punishment on behavior problems in early childhood | Dissertation |
| 99. | MacDonald (2013) | The privatization of public safety in urban neighborhoods: Do business improvement districts reduce violent crime among adolescents? | Not addressing predictor-outcome relationship |
| 100. | Madigan (2016) | Neighborhood Collective Efficacy Moderates the Association between Maternal Adverse Childhood Experiences and Marital Conflict. | No child externalizing outcomes addressed |
| 101. | Maimon (2009) | The circles of control: Integrating control and situational explanations of crime in the study of adolescents' violent encounters | Dissertation |
| 102. | Mason-Dorman (2014) | School district of philadelphia student achievement as related to 2008-2009 k-8 teachers' perceptions of major academic indicators | Dissertation |
| 103. | Matthews (2019) | Loneliness and Neighborhood Characteristics: A Multi-Informant, Nationally Representative Study of Young Adults. | No child externalizing outcomes addressed |
| 104. | Mayne (2022) | Association of Neighborhood Social Context and Perceived Stress Among Mothers of Young Children. | No child externalizing outcomes addressed |
| 105. | Mayne (2022) | Associations of Neighborhood Safety and Collective Efficacy with Dietary Intake among Preschool-Aged Children and Mothers. | No child externalizing outcomes addressed |
| 106. | McDonell (2011) | Construction and validation of an observational scale of neighborhood characteristics | No child externalizing outcomes addressed |
| 107. | McIntyre (2019) | The power of place: The impact of neighborhoods and racial socialization on academic outcomes | Dissertation |
| 108. | McNeeley (2018) | Lifestyle-Routine Activities, Neighborhood Context, and Ethnic Hate Crime Victimization. | No child externalizing outcomes addressed |
| 109. | Molnar (2016) | Neighborhood-level social processes and substantiated cases of child maltreatment. | No child externalizing outcomes addressed |
| 110. | Molnar (2005) | What girls need: Recommendations for preventing violence among urban girls in the US | Not a quantitative study design |
| 111. | Moulin (2017) | Correlates and predictors of well-being in Montreal | No child externalizing outcomes addressed |
| 112. | Nichter (2010) | Developing a smoke free household initiative: an Indonesian case study. | No child externalizing outcomes addressed |
| 113. | Nichter (2015) | Developing a smoke free homes initiative in Kerala, India. | No child externalizing outcomes addressed |
| 114. | O’Neal (2001) | Paths of resilience: A contextual-moderator analysis of exposure to community violence and behavioral functioning among inner city youth | Dissertation |
| 115. | Ohmer (2013) | Using Photovoice to Empower Youth and Adults to Prevent Crime. | No child externalizing outcomes addressed |
| 116. | Olamijuwon (2018) | Social cohesion and self-rated health among adults in South Africa: The moderating role of race. | Not focused on child-oriented outcomes |
| 117. | Pei (2022) | Changes of perceived Neighbourhood environment: A longitudinal study of collective efficacy among vulnerable families. | Not focused on child-oriented outcomes |
| 118. | Pratt (2020) | What Differences Do They See? Using Mixed Methods to Capture Adolescent Perceptions of Neighborhood Contexts. | No child externalizing outcomes addressed |
| 119. | Price-Wolf (2015) | Social support, collective efficacy, and child physical abuse: does parent gender matter? | No child externalizing outcomes addressed |
| 120. | Quinn (2011) | An ecological examination of psychological stress and asthma among low-income families in Chicago: Family, housing and neighborhood determinants | Dissertation |
| 121. | Quinones (2020) | The neighborhood-school spill-over: Middle and high school students' perceptions of racial/ethnic discrimination at the neighborhood and school level | Dissertation |
| 122. | Reboussin (2019) | Neighborhood context and transitions in marijuana use among urban young adults. | Not focused on child-oriented outcomes; no child externalizing outcomes addressed |
| 123. | Riley (2017) | Preparing for Disaster: a Cross-Sectional Study of Social Connection and Gun Violence. | Not focused on child-oriented outcomes |
| 124. | Rothman (2011) | Neighborhood-level factors associated with physical dating violence perpetration: results of a representative survey conducted in Boston, MA. | No child externalizing outcomes addressed |
| 125. | Sandel (2016) | Neighborhood-Level Interventions to Improve Childhood Opportunity and Lift Children Out of Poverty. | No child externalizing outcomes addressed |
| 126. | Sapouna (2010) | Collective efficacy in the school context: does it help explain victimization and bullying among Greek primary and secondary school students? | Not addressing predictor-outcome relationship |
| 127. | Schnellinger (2021) | Disorganization, communities, and prescription drugs: An investigation of the social context of non-medical use | Dissertation |
| 128. | Schnurr (2009) | Precursors to adolescents' dating violence perpetration and healthy romantic relationships | Dissertation |
| 129. | Schumann (2014) | Power differentials in bullying: individuals in a community context. | No child externalizing outcomes addressed |
| 130. | Sharkey (2015) | Violence, cognition, and neighborhood inequality in America | Not a peer-reviewed research publication |
| 131. | Showalter (2017) | Investigating the potentially protective effects of neighborhood processes in intimate partner violence | Not focused on child-oriented outcomes |
| 132. | Shuey (2018) | Neighborhood context and center-based child care use: Does immigrant status matter? | Not addressing predictor-outcome relationship; no child externalizing outcomes addressed |
| 133. | Simons (2011) | Errata:'Learning to be bad: adverse social conditions, social schemas, and crime' | No child externalizing outcomes addressed |
| 134. | Smith (2019) | Social learning, social disorganization, and psychological risk factors for criminal gangs in a British youth context | Not addressing predictor-outcome relationship |
| 135. | Soller (2014) | Legal cynicism and parental appraisals of adolescent violence | No child externalizing outcomes addressed |
| 136. | Spokane (2013) | Housing arrays following disasters: Social vulnerability considerations in designing transitional communities | No child externalizing outcomes addressed |
| 137. | Steinmetz-Wood (2017) | Is gentrification all bad? Positive association between gentrification and individual's perceived neighborhood collective efficacy in Montreal, Canada. | Not focused on child-oriented outcomes; no child externalizing outcomes addressed |
| 138. | Sternhal (2010) | Community violence and urban childhood asthma: a multilevel analysis. | No child externalizing outcomes addressed |
| 139. | Stoddard (2015) | Promoting Positive Future Expectations During Adolescence: The Role of Assets. | No child externalizing outcomes addressed |
| 140. | Suglia (2016) | Why the Neighborhood Social Environment Is Critical in Obesity Prevention. | No child externalizing outcomes addressed |
| 141. | Sullivan (2017) | Associations of neighborhood social environment attributes and physical activity among 9-11 year old children from 12 countries. | No child externalizing outcomes addressed |
| 142. | Taylor (2015) | 'A mother's gotta' do what she can': Neighborhood influences on the parenting strategies of mothers raising preadolescent children in high-risk environments | Dissertation |
| 143. | Tendulkar (2012) | Neighborhood influences on perceived social support among parents: Findings from the project on human development in Chicago neighborhoods | Not focused on child-oriented outcomes |
| 144. | Thaweekoon (2006) | Effects of exposure to community violence on adolescent adjustment problems | Dissertation |
| 145. | Theall (2009) | Social capital and the neighborhood alcohol environment. | Not focused on child-oriented outcomes; no child externalizing outcomes addressed |
| 146. | Thomas (2014) | Bad boys of bad odds? - race, context, and social influence: An investigation of youth violence in African-American boys | Dissertation |
| 147. | Vaeth PAC (2016) | Factors Associated with Depression Among Mexican Americans Living in U.S.-Mexico Border and Non-Border Areas. | Not focused on child-oriented outcomes; no child externalizing outcomes addressed |
| 148. | Vogel (2013) | Impulsivity, school context, and school misconduct | Not addressing predictor-outcome relationship |
| 149. | Walker (2016) | Impact of crime tolerance in low-income housing on neighbor cohesion and collective efficacy | No child externalizing outcomes addressed |
| 150. | Wang (2019) | The Relationship between Parental Perception of Neighborhood Collective Efficacy and Physical Violence by Parents against Preschool Children: A Cross-Sectional Study in a County of China. | No child externalizing outcomes addressed |
| 151. | Ware (2006) | Latina mothers' parenting and girls' anxiety and depression in an urban sample: Associations with ethnic identity and neighborhood context | Dissertation |
| 152. | Weatherburn (2006) | What Mediates the Macro-Level Effects of Economic and Social Stress on Crime? | No child externalizing outcomes addressed |
| 153. | Welch (2006) | Neighborhood, housing and women's health disparities. | Dissertation |
| 154. | Whipple (2021) | Expanding Collective Efficacy Theory to Reduce Violence Among African American Adolescents. | No child externalizing outcomes addressed |
| 155. | Wikström (2018) | Young people’s differential vulnerability to criminogenic exposure: Bridging the gap between people- and place-oriented approaches in the study of crime causation | No child externalizing outcomes addressed |
| 156. | Witherspoon (2008) | Positive neighborhood processes, structural disadvantage, and adolescent development | Dissertation |
| 157. | Witherspoon (2011) | An examination of social disorganization and pluralistic neighborhood theories with rural mothers and their adolescents | No child externalizing outcomes addressed |
| 158. | Witherspoon (2019) | Parenting within residential neighborhoods: A pluralistic approach with African American and Latino families at the center | No child externalizing outcomes addressed |
| 159. | Witherspoon (2019) | Parenting within residential neighborhoods: A pluralistic approach with African American and Latino families at the center | Duplicate that was not removed from initial  review |
| 160. | Wolf (2018) | Child maltreatment reporting in the general population: Examining the roles of community, collective efficacy, and adverse childhood experiences. | No child externalizing outcomes addressed |
| 161. | Wright (2015) | Intimate Partner Violence and Subsequent Depression: Examining the Roles of Neighborhood Supportive Mechanisms. | Not focused on child-oriented outcomes; no child externalizing outcomes addressed |
| 162. | Zimmerman (2015) | Investigating the role of neighborhood youth organizations in preventing adolescent violent offending: Evidence from Chicago | No child externalizing outcomes addressed |
| 163. | Zuberi (2016) | Neighborhoods and parenting: Assessing the influence of neighborhood quality on the parental monitoring of youth | No child externalizing outcomes addressed |
| 164. | N/A | Abstracts from the 2013 Annual Scientific Meeting of the American Psychosomatic Society | Not a peer-reviewed research publication |

S1 Table 2. Studies with full text review that were excluded (N=113) and included (N=17) (Total N=130)

|  | Anderson (2015) | Ecology matters: Neighborhood differences in the protective role of self-control and social support for adolescent antisocial behavior. | No child externalizing outcomes addressed |
| --- | --- | --- | --- |
|  | Azrael (2009) | Creating a youth violence data system for Boston, Massachusetts | No child externalizing outcomes addressed |
|  | Baishya (2023) | Urban neighbourhood elements that influence psychoactive substance use among populations with adverse childhood experiences: a scoping review protocol | No child externalizing outcomes addressed |
|  | Banyard (2019) | 'What would the neighbors do?' measuring sexual and domestic violence prevention social norms among youth and adults | No child externalizing outcomes addressed |
|  | Barnhart (2018) | Perceptions of collective efficacy among single mothers: Insights for conceptualization and measurement | No child externalizing outcomes addressed |
|  | Bauer (2022) | The Protective Effect of Neighbourhood Collective Efficacy On Family Violence and Youth Antisocial Behaviour in Two South Korean Prospective Longitudinal Cohorts. | No child externalizing outcomes addressed |
|  | Beck (2012) | Strategies for Preventing Neighborhood Violence: Toward Bringing Collective Efficacy into Social Work Practice. | Not a quantitative study design |
|  | Betancourt (2014) | Context matters: community characteristics and mental health among war-affected youth in Sierra Leone | Included |
|  | Bhargava (2017) | Examining socio-cultural and neighborhood factors associated with trajectories of Mexican-origin mothers’ education-related involvement | No child externalizing outcomes addressed |
|  | Brady (2006) | Lifetime community violence exposure and health risk behavior among young adults in college. | No child externalizing outcomes addressed |
|  | Browning (2005) | Sexual initiation in early adolescence: The nexus of parental and community control | No child externalizing outcomes addressed |
|  | Browning (2004) | Neighborhood context and racial differences in early adolescent sexual activity. | No child externalizing outcomes addressed |
|  | Browning (2014) | Collective Efficacy and the Contingent Consequences of Exposure to Life-Threatening Violence | Included |
|  | Browning (2015) | Neighborhoods and adolescent health-risk behavior: an ecological network approach. | No child externalizing outcomes addressed |
|  | Byck (2015) | Effect of housing relocation and neighborhood environment on adolescent mental and behavioral health | Not addressing predictor-outcome relationship |
|  | Carter (2024) | Land-use filtering for nonstationary spatial prediction of collective efficacy in an urban environment | Not focused on child-oriented outcomes; no child externalizing outcomes addressed |
|  | Caron (2012) | Prevalence of psychological distress and mental disorders, and use of mental health services in the epidemiological catchment area of Montreal South-West | Not focused on child-oriented outcomes |
|  | Castillo (2020) | Child externalizing behavior in context: Associations of mother nonstandard work, parenting, and neighborhoods | Included |
|  | Chang (2015) | Direct and indirect effects of neighborhood characteristics on the perpetration of dating violence across adolescence. | No child externalizing outcomes addressed |
|  | Chang (2018) | Maternal monitoring knowledge change and adolescent externalizing behaviors in low-income African American and Latino families | Not addressing predictor-outcome relationship |
|  | Cho (2017) | Self-control and risky lifestyles in context: Cross-level integration between opportunity and collective efficacy in the study of peer victimization among South Korean youth | No child externalizing outcomes addressed |
|  | Choi (2021) | Neighborhood disadvantage, childhood adversity, bullying victimization, and adolescent depression: A multiple mediational analysis. | No child externalizing outcomes addressed |
|  | Coley (2004) | Out-of-school care and problem behavior trajectories among low-income adolescents: individual, family, and neighborhood characteristics as added risks. | No child externalizing outcomes addressed |
|  | Cooley-Strickland (2009) | Community violence and youth: Affect, behavior, substance use, and academics | No child externalizing outcomes addressed |
|  | Donnelly (2016) | Cohesive Neighborhoods Where Social Expectations Are Shared May Have Positive Impact On Adolescent Mental Health. | No child externalizing outcomes addressed |
|  | Drinkard (2017) | Predicting prosociality among urban adolescents: Individual, family, and neighborhood influences | No child externalizing outcomes addressed |
|  | Du (2020) | Family functioning and adolescent behavior problems: A moderated mediation model of caregiver depression and neighborhood collective efficacy | Not addressing predictor-outcome relationship |
|  | Ellis (2015) | Protective factors for violence perpetration in Somali young adults: The role of community belonging and neighborhood cohesion | No child externalizing outcomes addressed |
|  | Emery (2015) | Neighborhood informal social control and child maltreatment: A comparison of protective and punitive approaches | Included |
|  | Emory (2008) | Neighborhood social processes and academic achievement in elementary school | No child externalizing outcomes addressed |
|  | Ernestus (2015) | Patterns of risk and resilience in African American and Latino youth | No child externalizing outcomes addressed |
|  | Fagan (2014) | The protective effects of neighborhood collective efficacy on adolescent substance use and violence following exposure to violence. | No child externalizing outcomes addressed |
|  | Fagan (2015) | A multi-level analysis of the impact of neighborhood structural and social factors on adolescent substance use. | No child externalizing outcomes addressed |
|  | Fan (2012) | Family functioning as a mediator between neighborhood conditions and children's health: evidence from a national survey in the United States. | No child externalizing outcomes addressed |
|  | Fleckman (2022) | Neighborhood matters: Neighborhood violence, collective efficacy, and social emotional development in early childhood | No child externalizing outcomes addressed |
|  | Fleckman (2024) | From neighborhood to household: connections between neighborhood vacant and abandoned property and family violence | No child externalizing outcomes addressed |
|  | Gard (2022) | Deadly gun violence, neighborhood collective efficacy, and adolescent neurobehavioral outcomes. | Not addressing predictor-outcome relationship |
|  | Guo (2024) | The prevalence of cyberbullying and the association between cyberbullying emotional exhaustion and perception of collective efficacy among Chinese college students | No child externalizing outcomes addressed |
|  | Han (2019) | Neighborhood predictors of bullying perpetration and victimization trajectories among South Korean adolescents. | No child externalizing outcomes addressed |
|  | Hardi (2024) | Childhood adversity and adolescent mental health: Examining cumulative and specificity effects across contexts and developmental timing | Included |
|  | Held (2020) | Predictors of Latinx youth health and emotional well-being: Social determinants of health perspective | No child externalizing outcomes addressed |
|  | Huang (2014) | The location of placement and juvenile delinquency: Do neighborhoods matter in child welfare? | No child externalizing outcomes addressed |
|  | Ichikawa (2017) | It takes a village: Fixed-effects analysis of neighborhood collective efficacy and children's development | Included |
|  | Jackson (2016) | The role of immigrant concentration within and beyond residential neighborhoods in adolescent alcohol use | No child externalizing outcomes addressed |
|  | Jackson (2014) | Social and socio-demographic neighborhood effects on adolescent alcohol use: a systematic review of multi-level studies. | No child externalizing outcomes addressed |
|  | Jackson (2016) | The role of neighborhood disadvantage, physical disorder, and collective efficacy in adolescent alcohol use: a multilevel path analysis. | No child externalizing outcomes addressed |
|  | Jain (2013) | Behavioral adaptation among youth exposed to community violence: a longitudinal multidisciplinary study of family, peer and neighborhood-level protective factors. | Not addressing predictor-outcome relationship |
|  | Jensen (2020) | Stepfamily Processes and Youth Adjustment: The Role of Perceived Neighborhood Collective Efficacy. | No child externalizing outcomes addressed |
|  | Johnson (2016) | Caregiver and adolescent discrepancies in perceptions of violence and their associations with early adolescent aggression | No child externalizing outcomes addressed |
|  | Kim (2010) | Influence of neighbourhood collective efficacy on adolescent sexual behaviour: variation by gender and activity participation. | No child externalizing outcomes addressed |
|  | Kim (2024) | Does High Self-Control Accelerate Epigenetic Aging in Low-Income Adolescents? | Not addressing predictor-outcome relationship |
|  | Kofler (2022) | Psychopathic Traits and Conduct Problems in Children: Effects of Collective Efficacy, Heart Rate, and Sex | Included |
|  | Krishnaukumar (2014) | Multilevel and cross-level effects of neighborhood and family influences on children's behavioral outcomes in Trinidad and Tobago: the intervening role of parental control. | No child externalizing outcomes addressed |
|  | Kronaizl (2023) | Perceived neighbourhood collective efficacy and adolescent health determinants: Investigating outdoor play as a mediator. | No child externalizing outcomes addressed |
|  | Kurlycheck (2012) | Protection from risk: Exploration of when and how neighborhood-level factors can reduce violent youth outcomes | No child externalizing outcomes addressed |
|  | Lackey (2018) | A model of rural delinquency: Collective efficacy in rural schools | No child externalizing outcomes addressed |
|  | Lee (2021) | Relationships between neighborhood collective efficacy and adolescent suicidal ideation. | No child externalizing outcomes addressed |
|  | Lee (2021) | Relationships between neighborhood collective efficacy and adolescent suicidal ideation | Duplicate that was not removed from initial review |
|  | Leventhal (2000) | The neighborhoods they live in: the effects of neighborhood residence on child and adolescent outcomes. | Not a quantitative study design |
|  | Leventhal (2003) | Moving on up: Neighborhood effects on children and families | Not a peer-reviewed research publication |
|  | Lew (2022) | The Longitudinal Impact of Maternal Depression and Neighborhood Social Context on Adolescent Mental Health. | No child externalizing outcomes addressed |
|  | Lindstrom Johnson (2011) | Examining the link between neighborhood context and parental messages to their adolescent children about violence. | No child externalizing outcomes addressed |
|  | Liu (2016) | Risk and protective factors for comorbid internalizing and externalizing problems among economically disadvantaged African American youth | Included |
|  | Liu (2017) | Risk and protective factors for comorbid internalizing and externalizing problems among economically disadvantaged African American youth. | Duplicate that was not removed from initial  review |
|  | Longhi (2021) | Community-wide resilience mitigates adverse childhood experiences on adult and youth health, school/work, and problem behaviors. | No child externalizing outcomes addressed |
|  | Ma (2016) | Neighborhood and parenting both matter: The role of neighborhood collective efficacy and maternal spanking in early behavior problems | Included |
|  | Ma (2017) | Longitudinal Associations of Neighborhood Collective Efficacy and Maternal Corporal Punishment with Behavior Problems in Early Childhood | Included |
|  | Ma (2018) | Does Race/Ethnicity Moderate the Associations between Neighborhood and Parenting Processes on Early Behavior Problems? | Included |
|  | Ma (2018) | Neighborhood collective efficacy, parental spanking, and subsequent risk of household child protective services involvement. | No child externalizing outcomes addressed |
|  | Madigan (2017) | Trajectories of maternal depressive symptoms in the early childhood period and family-wide clustering of risk. | No child externalizing outcomes addressed |
|  | Maimon (2012) | Underage drinking, alcohol sales and collective efficacy: Informal control and opportunity in the study of alcohol use. | No child externalizing outcomes addressed |
|  | Maimon (2010) | Collective efficacy, family attachment, and urban adolescent suicide attempts. | No child externalizing outcomes addressed |
|  | Maimon (2010) | Unstructured socializing, collective efficacy, and violent behavior among urban youth | No child externalizing outcomes addressed |
|  | Maimon (2012) | Adolescents' violent victimization in the neighborhood: Situational and contextual determinants | No child externalizing outcomes addressed |
|  | Martinez-Torteya (2015) | Profiles of adaptation among child victims of suspected maltreatment | Not addressing predictor-outcome relationship |
|  | Martoccio (2022) | Preventing early harsh parenting and toddler behavior problems: The role of neighborhood collective efficacy among low-income Latine families. | Not addressing predictor-outcome relationship |
|  | McDermott (2017) | Self‐control and adolescent internalizing and externalizing problems: Neighborhood‐based differences | Not addressing predictor-outcome relationship |
|  | McDonell (2015) | Strong Communities for Children: Results of a multi-year community-based initiative to protect children from harm. | No child externalizing outcomes addressed |
|  | Meier (2008) | Impulsive and callous traits are more strongly associated with delinquent behavior in higher risk neighborhoods among boys and girls. | No child externalizing outcomes addressed |
|  | Molnar (2004) | Neighborhood predictors of concealed firearm carrying among children and adolescents: results from the project on human development in Chicago neighborhoods. | No child externalizing outcomes addressed |
|  | Moren-Cross (2006) | Perceived Neighborhood Characteristics and Problem Behavior Among Disadvantaged Children | Included |
|  | Niwa (2021) | The spaces between: Parents' perceptions of neighborhood cohesion and child well-being | No child externalizing outcomes addressed |
|  | Odgers (2009) | The Protective Effects of Neighborhood Collective Efficacy on British Children Growing Up in Deprivation: A Developmental Analysis | Included |
|  | O’Brien (2013) | Broken windows and low adolescent prosociality: not cause and consequence, but co-symptoms of low collective efficacy. | No child externalizing outcomes addressed |
|  | Orihuela (2020) | Neighborhood disorder, family functioning, and risky sexual behaviors in adolescence | No child externalizing outcomes addressed |
|  | Pei (2022) | Neighborhood influences on early childhood behavioral problems: Child maltreatment as a mediator | Included |
|  | Pei (2024) | The effects of two types of neighborhood factors on trajectory of internalizing and externalizing symptoms from early childhood to adolescence | Included |
|  | Pinchak (2022) | Racial Inequalities in Adolescents' Exposure to Racial and Socioeconomic Segregation, Collective Efficacy, and Violence. | No child externalizing outcomes addressed |
|  | Prince (2019) | Collective Efficacy as a Key Context in Neighborhood Support for Urban Youth. | No child externalizing outcomes addressed |
|  | Puleo (2024) | Neighborhood Collective Efficacy: A Longitudinal Social Determinant of Access to Medical Care Among Recently Immigrated Latina Young Adults | Not focused on child-oriented outcomes |
|  | Quinn (2010) | Parent perceptions of neighborhood stressors are associated with general health and child respiratory health among low-income, urban families. | No child externalizing outcomes addressed |
|  | Rabinowitz (2020) | Neighborhood Profiles and Associations with Coping Behaviors among Low-Income Youth. | No child externalizing outcomes addressed |
|  | Rankin (2002) | Social contexts and urban adolescent outcomes: The interrelated effects of neighborhoods, families, and peers on African-American youth | No child externalizing outcomes addressed |
|  | Riina (2019) | Neighborhood qualities and parenting among mothers with young children: Variation by relationship status | No child externalizing outcomes addressed |
|  | Rivera (2022) | The role of neighborhood collective efficacy in moderating associations between adversity and internalizing and externalizing problems in adolescents. | Not addressing predictor-outcome relationship |
|  | Sasser (2019) | A longitudinal investigation of protective factors for bereaved maltreated youth. | Not addressing predictor-outcome relationship |
|  | Schmidt (2016) | The mediating effect of future expectations on the relationship between neighborhood context and adolescent bullying perpetration | No child externalizing outcomes addressed |
|  | Schmidt (2020) | Do changes in neighborhood social context mediate the effects of the moving to opportunity experiment on adolescent mental health? | Not addressing predictor-outcome relationship |
|  | Schnurr (2013) | The impact of collective efficacy on risks for adolescents' perpetration of dating violence. | Not addressing predictor-outcome relationship |
|  | Sharkey (2006) | Navigating Dangerous Streets: The Sources and Consequences of Street Efficacy | No child externalizing outcomes addressed |
|  | Sharma (2019) | Protective Factors Buffer Life Stress and Behavioral Health Outcomes among High-Risk Youth. | Not addressing predictor-outcome relationship |
|  | Shuey (2017) | Pathways of risk and resilience between neighborhood socioeconomic conditions and parenting | No child externalizing outcomes addressed |
|  | Simons (2011) | Learning to be bad: Adverse social conditions, social schemas, and crime | No child externalizing outcomes addressed |
|  | Spilsbury (2022) | 'A rising tide floats all boats': The role of neighborhood collective efficacy in responding to child maltreatment | No child externalizing outcomes addressed |
|  | Sterrett-Hong (2023) | Genetic Risk, Neighborhood Characteristics, and Behavioral Difficulties Among African American Adolescents Living in Very Low-Income Neighborhoods | Included |
|  | Stritzel (2022) | Peer and Community Influences on Adolescent Substance Use in the Context of Adverse Childhood Experiences. | No child externalizing outcomes addressed |
|  | Stults (2021) | The role of neighborhood context in the relationship between parenting effectiveness and self-control | No child externalizing outcomes addressed |
|  | Sullivan (2014) | Individual, social, and neighborhood influences on the launch of adolescent antisocial behavior | No child externalizing outcomes addressed |
|  | Takakura (2019) | The Relative Association of Collective Efficacy in School and Neighborhood Contexts With Adolescent Alcohol Use. | No child externalizing outcomes addressed |
|  | Thomas (2016) | It's in my hood: Understanding African American boys’ perception of safety in their neighborhoods | No child externalizing outcomes addressed |
|  | Tompsett (2016) | Peer delinquency and where adolescents spend time with peers: Mediation and moderation of home neighborhood effects on self‐reported delinquency | No child externalizing outcomes addressed |
|  | Tompsett (2014) | Travel beyond the home neighborhood for delinquent behaviors: moderation of home neighborhood influences. | No child externalizing outcomes addressed |
|  | Vaughn (2023) | Preschoolers’ Psychosocial Development, Parents, and Neighborhoods: Towards an Integrative Approach for Immigrant Families | No child externalizing outcomes addressed |
|  | Wadsworth (2020) | Reducing the Biological and Psychological Toxicity of Poverty-related Stress: Initial Efficacy of the BaSICS Intervention for Early Adolescents. | No child externalizing outcomes addressed |
|  | Wang (2020) | Long-term Neighborhood Effects on Adolescent Outcomes: Mediated through Adverse Childhood Experiences and Parenting Stress | Included |
|  | Wang (2021) | The longitudinal influences of adverse childhood experiences and positive childhood experiences at family, school, and neighborhood on adolescent depression and anxiety. | No child externalizing outcomes addressed |
|  | Wang (2019) | Social Cohesion, Neighborhood Collective Efficacy, and Adolescent Subjective Well-being in Urban and Rural Taiwan. | No child externalizing outcomes addressed |
|  | Wei (2021) | Chinese American adolescents’ academic performance and psychological adjustment: The role of neighborhood and family | No child externalizing outcomes addressed |
|  | Weinberger (2023) | Developmental trajectories of conduct problems across racial/ethnic identity and neighborhood context: A systematic review | No child externalizing outcomes addressed |
|  | Wikström (2016) | Social disadvantage and crime: A criminological puzzle | No child externalizing outcomes addressed |
|  | Wilkinson (2019) | Disrupting the link between maltreatment and delinquency: how school, family, and community factors can be protective. | No child externalizing outcomes addressed |
|  | Witherspoon (2014) | Early adolescent perceptions of neighborhood: Strengths, structural disadvantage, and relations to outcomes | No child externalizing outcomes addressed |
|  | Xue (2005) | Neighborhood residence and mental health problems of 5- to 11-year-olds. | No child externalizing outcomes addressed |
|  | Yonas (2010) | Perceptions of neighborhood collective efficacy moderate the impact of maltreatment on aggression. | Not addressing predictor-outcome relationship |
|  | Yoshizawa (2009) | [Effects of neighborhood collective efficacy and violence on antisocial behavior: dual mediation of socialization and routine activities]. | Not focused on child-oriented outcomes |
|  | Yoshizawa (2020) | Cross-cultural protective effects of neighborhood collective efficacy on antisocial behaviors: Mediating role of social information processing | No child externalizing outcomes addressed |
|  | Zhen-Duan (2023) | Mexican-origin parent and child reported neighborhood factors and youth substance use | No child externalizing outcomes addressed |
|  | Zimmerman (2019) | Neighborhood through a familial lens: Examining the intergenerational transmission of collective efficacy | No child externalizing outcomes addressed |
|  | Zolotor (2006) | Social capital, family violence, and neglect. | No child externalizing outcomes addressed |
|  | Zuberi (2017) | Child health in low‐income neighborhoods: The unexpected relationship with neighborhood disorder and other aspects of distress | No child externalizing outcomes addressed |
